# IL‐10‐ and IL‐13‐Biased T Cell Responses to SARS‐CoV‐2 Vaccination in Diabetes

**DOI:** 10.1002/eji.70112

**Published:** 2025-12-09

**Authors:** Emma M. Jones, Caren Sourij, Martin Stradner, Peter Schlenke, Nazanin Sereban, Othmar Moser, Rachael Quinlan, Charlotte‐Eve Short, Benjamin H. L. Harris, Michael Fertleman, Graham P. Taylor, Nick Oliver, Harald Sourij, Margarita Dominguez‐Villar

**Affiliations:** ^1^ Department of Infectious Disease Faculty of Medicine Imperial College London London UK; ^2^ Division of Cardiology Medical University of Graz Austria; ^3^ Division of Rheumatology and Immunology Medical University of Graz Austria; ^4^ Department of Blood Group Serology and Transfusion Medicine Medical University of Graz Austria; ^5^ University of Bayreuth Bayreuth Germany; ^6^ Interdisciplinary Metabolic Medicine Trials Unit Division of Endocrinology and Diabetology Medical University of Graz Austria; ^7^ Imperial College NHS Trust London UK; ^8^ Department of Bioengineering Faculty of Engineering Imperial College London London UK; ^9^ Department of Metabolism Digestion and Reproduction Faculty of Medicine Imperial College London London UK

**Keywords:** vaccination, diabetes, type 1 diabetes, T cells, memory, SARS‐CoV‐2

## Abstract

Type 1 and type 2 diabetes are associated with increased severity and mortality from respiratory virus infections. Vaccination in the general population significantly reduces the risk of severe respiratory viral infection and triggers a strong, polyfunctional, and lasting T cell response in healthy individuals. However, vaccine effectiveness in people with type 1 diabetes is unclear. Here, we studied the magnitude and functional characteristics of vaccine‐specific CD4^+^ and CD8^+^ T cell responses to vaccination in people with type 1 and type 2 diabetes and compared them to those of people living without diabetes, using the severe acute respiratory syndrome coronavirus 2 (SARS‐CoV‐2) vaccine as a model. We found defects in both CD4^+^ and CD8^+^ T cell memory maintenance and the functionality of the vaccine‐specific T cells in people with diabetes compared with people without. In those individuals with type 1 and type 2 diabetes who harbored detectable vaccine‐specific T cells, they displayed an unfocused, tolerogenic phenotype characterized by increased expression of IL‐10 and IL‐13 compared with people without diabetes. These results have implications for vaccination strategies for people with diabetes.

## Graphical abstract

How the immune system of people with diabetes responds to vaccination against respiratory viruses is not well understood. Using activation‐induced markers to identify, quantify, and functionally characterize Spike‐specific CD4+ and CD8+ T cells after SARS‐CoV‐2 vaccination, we show defects in T cell memory maintenance and an IL‐10 and IL‐13‐dominated T cell response in people with diabetes, indicating an unfocused, tolerogenic phenotype.

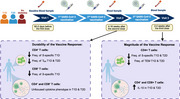

**Abbreviations**
IFNγ: interferon gamma
IL‐13: interleukin 13
IL‐17a: interleukin 17a
IL‐2: interleukin 2
IL‐21: interleukin 21
ND: no diabetes
PBMC: peripheral blood mononuclear cells
SARS‐CoV‐2: severe acute respiratory syndrome coronavirus 2
T1D: type 1 diabetes
T2D: type 2 diabetes
TNF: tumor necrosis factor

## 1 Introduction

People with type 1 (T1D) and type 2 (T2D) diabetes have a higher susceptibility to, and a more severe pathology from, respiratory viral infections [1, 2]. In prospective cohort studies, people with T1D and T2D have an odds ratio of 3.35 and 3.42, respectively, for severe illness after severe acute respiratory syndrome coronavirus 2 (SARS‐CoV‐2) infection compared with people without diabetes [2]. As such, people with diabetes are often placed in high‐risk categories for vaccination prioritization strategies [3, 4]. In addition, despite limited understanding of the immunological response to vaccines in people with diabetes, the same vaccination protocol against respiratory viruses as the one followed for the general population is recommended for patients with diabetes, including for Influenza and SARS‐CoV‐2 [3, 4, 5]. There is limited information about the durability, magnitude, and nature of the cellular immune response to vaccination in people with diabetes, and previous studies have reported conflicting results. Most studies on hepatitis B vaccination have demonstrated reduced immunogenicity in people with diabetes [6]. Data on response to other vaccines, including those against the Influenza virus, appear mostly inconclusive, with contradicting findings regarding the humoral response in people with diabetes, and evidence suggesting that antibody titers are not a good correlate of protection against the Influenza virus in this study population [4]. Regarding SARS‐CoV‐2 vaccination, some evidence suggests that CD4^+^ T cell cytokine responses and neutralizing antibody titers are lower in people with T2D with higher glycemic exposure compared with those with glucose levels close to target and people without diabetes [7], while other investigations did not observe differences in antibody titers [8]

Using SARS‐CoV‐2 vaccination as a model, we have dissected the cellular immune response to vaccination in people with T1D or T2D by examining both T cell memory durability and functionality after the full vaccination protocol and the magnitude and nature of the recall response after a booster dose of SARS‐CoV‐2 vaccine. Participants with diabetes displayed an impairment in the durability of the S‐specific T cells after the full vaccination schedule. In those diabetes participants where S‐specific T cells were detected, the response was unfocused, with the expected expression of IFNγ, TNF, and IL‐2, alongside a significant increase in the expression of the Th2 cytokine IL‐13 in S‐specific CD4^+^ and CD8^+^ T cells from both diabetes groups. Recall T cell responses to a booster dose of the SARS‐CoV‐2 vaccine were severely impaired in participants with diabetes compared with those without diabetes. While more than 80% of normoglycemic controls harbored S‐specific CD4^+^ T cells, only 30% of participants with T1D and about 20% of those with T2D had detectable S‐specific CD4^+^ T cells. In those individuals where S‐specific CD4^+^ T cells were identified, a significant increase in IL‐13‐ and IL‐10‐producing cells was observed. In those participants with T1D or T2D in which S‐specific CD8^+^ T cells were detected, they also displayed a tolerogenic phenotype, with IL‐10 being co‐produced with most Tc‐specific cytokines. These results suggest defects in both vaccine‐specific T cell memory maintenance and alterations in the nature of the vaccine‐specific T cell response in diabetes and have implications for vaccination strategies in people with diabetes.

## 2 Results

### 2.1 Vaccine‐Specific CD4^+^ and CD8^+^ T Cell Memory Is Impaired in T1D

**FIGURE 1:**
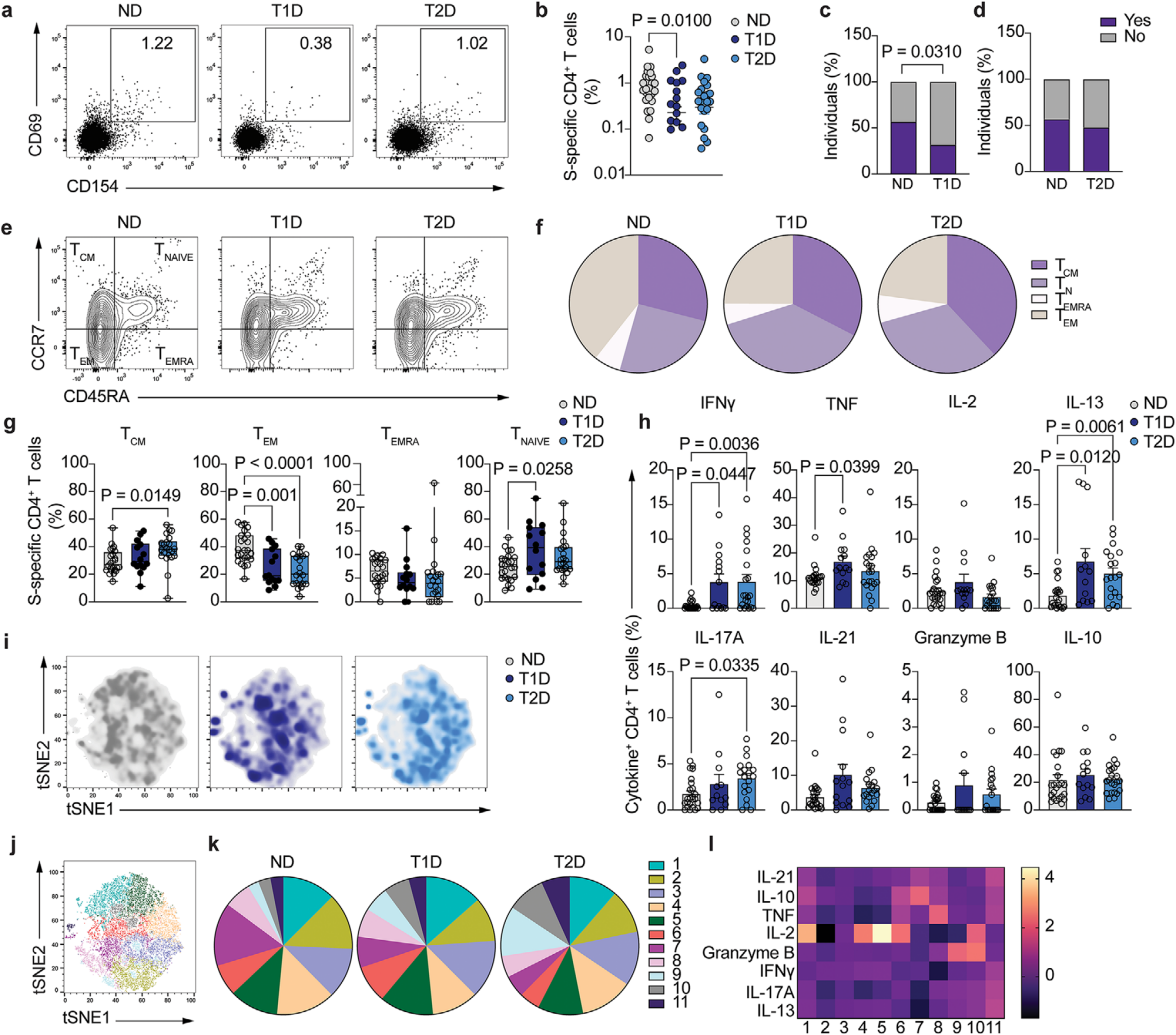
Vaccine‐specific CD4^+^ T cell maintenance. (**A**) Representative dot plot of S‐specific CD4^+^ T cells (CD154^+^CD69^+^) after stimulation with SARS‐CoV‐2 spike peptide pool in people without diabetes (ND, left), T1D (middle), and T2D (right) participants. (**B**) Summary of frequency of S‐specific CD4^+^ T cells from total CD4^+^ T cells (*n =* 24 ND, *n* = 14 T1D, and *n* = 22 T2D). (**C**) Percentage of individuals i*n* whom S‐specific CD4^+^ T cells were (purple) or not (grey) detected in ND controls (*n* = 44) and T1D patients (*n* = 44). (**D**) Percentage of individuals in whom S‐specific CD4^+^ T cells were (purple) or not (grey) detected in ND controls (*n =* 44) and T2D patients (*n =* 46). (**E**) Representative dot plot of the expression of CD45RA and CCR7 in S‐specific CD4^+^ T cells from ND (left), T1D (middle), and T2D (right) participants. (**F**) Percentage of S‐specific CD4^+^ T cells from ND controls (left, *n =* 24), T1D (middle, *n =* 14), and T2D (right, *n =* 22) with naïve, memory, central memory, or effector memory cells re‐expressing CD45RA phenotype. (**G**) Summary of S‐specific CD4^+^ T cells from ND controls (*n =* 24), T1D (*n =* 14), and T2D (*n =* 22) patients with naïve, memory, central memory, or effector memory cells re‐expressing CD45RA phenotype. (**H**) Summary of the frequency of cytokine‐expressing S‐specific CD4^+^ T cells from ND (*n =* 21‐24), T1D (*n =* 14), and T2D (*n =* 19‐22) participants. (**I**) tSNE projection of S‐specific CD4^+^ T cells in ND (left), T1D (middle), and T2D (right) participants. (**J**) tSNE projection of S‐specific CD4^+^ T cell clusters using cells from the 3 study groups. (**K**) Percentage of S‐specific CD4^+^ T cells in each identified cluster for ND (left), T1D (middle), and T2D (right) individuals. (**L**) Heatmap of geometric mean fluorescence intensity displayed as modified *z*‐scores using median values (row‐scaled). One‐way ANOVA with Tukey's correction for multiple comparisons (**B**, **G**), two‐tailed Fisher's exact test (**C**, **D**), Kruskal–Wallis test with correction for multiple comparisons (**H**).

To examine the durability and quality of vaccine‐specific T cell immune memory in patients with diabetes, we analyzed peripheral blood mononuclear cells (PBMC) of participants with either T1D or T2D and control individuals without diabetes (ND). We investigated the immunogenicity of SARS‐CoV‐2 vaccines 1–4 months after completing the full course of vaccination (two doses) to determine whether people with diabetes maintained a similar magnitude and functional characteristics of vaccine‐specific cellular responses to people without diabetes. We used a library of overlapping peptides covering the whole SARS‐CoV‐2 Wuhan wild‐type spike protein (S) and activation‐induced markers to identify vaccine‐specific CD4^+^ and CD8^+^ T cells after a short stimulation of total PBMC with the peptide library (Figure 1A). Participants with T1D, but not with T2D, showed a significant decrease in the frequency of S‐specific CD4^+^ T cells, identified as CD154^+^CD69^+^, compared with people without diabetes (approximately 2.7‐fold decrease in T1D compared with the no diabetes group, Figure 1B). More strikingly, the number of people without diabetes in which we could detect S‐specific CD4^+^ T cells at the time point examined was significantly higher than that of T1D participants (25 out of 44 ND and 14 out of 44 T1D, *p* = 0.031), while the T2D group showed a comparable number of participants harboring S‐specific CD4^+^ T cells as ND (Figure 1C). S‐specific CD4^+^ T cells from the ND group displayed enhanced antigen experience as compared with T1D, with a higher proportion of memory T cells compared with diabetes groups and a lower proportion of naïve T cells (T_NAIVE_) compared with participants with T1D (Figures 1D,E). Thus, those with T1D and T2D displayed a significant decrease in the frequency of effector memory cells (T_EM_) compared with ND controls, and T1D participants further displayed a significant increase in naïve S‐specific CD4^+^ T cells compared with ND (Figure 1F). These results were not due to a difference in the distribution of memory CD4^+^ T cells among the three study groups, as no differences were observed in the frequency of central memory (T_CM_), effector memory (T_EM_), and effector memory re‐expressing CD45RA (T_EMRA_) cells in total CD4^+^ T cells among groups (Figure S1). These results suggest that in people with T1D and T2D, S‐specific memory CD4^+^ T cell responses are reduced compared with those of people without diabetes, and upon antigen challenge, S‐specific CD4^+^ T cells are activated mostly from the naïve T cell pool.

The production of multiple cytokines by T cells has been associated with productive immune responses to vaccines [9, 10, 11], so we further characterized the polyfunctionality of S‐specific CD4^+^ T cells by determining the expression of cytokines associated with an optimal response to vaccination, including IFNγ, TNF, and IL‐2 [12, 13]. We also included in the analysis other cytokines that are typically secreted by various helper T cell populations, such as IL‐13, IL‐17A, IL‐21, granzyme B, and the anti‐inflammatory cytokine IL‐10 (Figure 1G). A significantly increased proportion of S‐specific CD4^+^ T cells produced IFNγ and TNF in participants with T1D compared with ND, and S‐specific CD4^+^ T cells from participants with T2D displayed an increased expression of IFNγ and IL‐17A. However, common to both T1D and T2D S‐specific CD4^+^ T cell responses was the increased secretion of the Th2‐related cytokine IL‐13, suggesting a Th2‐biased vaccine response. In order to better understand the heterogeneity in the functionality of S‐specific CD4^+^ T cells in the study groups, we generated tSNE plots using all concatenated S‐specific CD4^+^ T cells from those individuals in which they were detected. Apparent differences could be observed in the tSNE plot from ND participants compared with those of individuals with diabetes, with more similarities between the plots from T1D and T2D (Figure 1H). Unsupervised clustering analysis identified 11 different clusters of S‐specific CD4^+^ T cells (Figure 1I; Figure S2), and the distribution of cells within such clusters did not vary significantly between ND and participants with diabetes (Figure 1J), with only people with T2D displaying a modest decrease in the frequency of cells in Cluster 1 and a significant increase in the frequency of cells in clusters 9 and 11 compared with ND (Figure S3; Table S2). Cytokine characterization of each cluster (Figure 1K) suggested that cluster 1 contained cells with an IL‐2 and IL‐10 production profile, cluster 9 mainly produced granzyme B, and cluster 11 was a population with a heterogeneous pattern of cytokine secretion. These results suggest that S‐specific CD4^+^ T cells in people with diabetes display an unfocused cytokine phenotype with no particular enrichment of a Th2‐like population, but rather a global increase in IL‐13 production and no major changes in the distribution of cells within the identified clusters among study groups.

**FIGURE 2:**
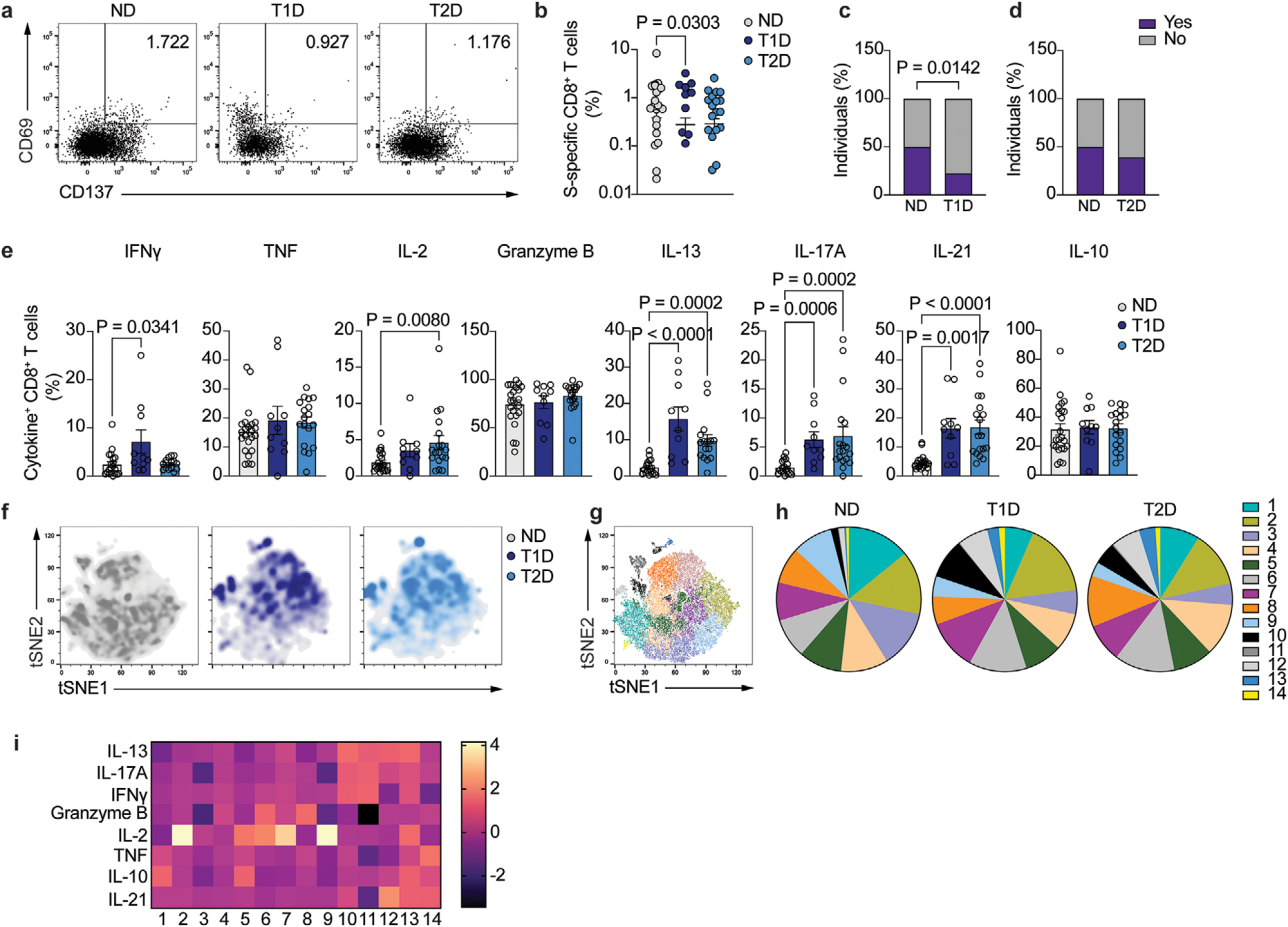
Vaccine‐specific CD8^+^ T cell maintenance. (**A**) Representative dot plot of S‐specific CD8^+^ T cells (CD137^+^CD69^+^) after stimulation with SARS‐CoV‐2 spike peptide pool in ND (left), T1D (middle), and T2D (right) participants. **(B)** Summary of frequency of S‐specific CD8^+^ T cells from total CD8^+^ T cells (*n =* 21 ND, *n =* 10 T1D, and *n =* 18 T2D). **(C)** Percentage of individuals in whom S‐specific CD8^+^ T cells were (purple) or not (grey) detected in ND controls (*n =* 44) and T1D participants (*n =* 44). **(D)** Percentage of individuals in whom S‐specific CD8^+^ T cells were (purple) or not (grey) detected in ND controls (*n =* 44) and T2D participants (*n =* 46). **(E)** Summary of the frequency of cytokine‐expressing S‐specific CD8^+^ T cells from ND individuals (*n =* 18–22), T1D (*n =* 10), and T2D (*n =* 16–18) participants. **(F)** tSNE projection of S‐specific CD8^+^ T cells from ND controls (left), T1D (middle), and T2D (right) patients. **(G)** tSNE projection of S‐specific CD4^+^ T cell clusters using cells from the 3 study groups. **(H)** Percentage of S‐specific CD8^+^ T cells in each identified cluster for ND (left), T1D (middle), and T2D (right) participants. **(I)** Heatmap of geometric mean fluorescence intensity displayed as modified *z*‐scores using median values (row‐scaled). One‐way ANOVA with Tukey's correction for multiple comparisons (**B**), two‐tailed Fisher's exact test (**C**, **D**), Kruskal–Wallis test with correction for multiple comparisons (**E**).

We went on to quantify and functionally characterize CD8^+^ T cell memory to vaccination. S‐specific CD8^+^ T cells, identified as CD137^+^CD69^+^ (Figure 2A), were significantly reduced in frequency in participants with T1D, but not in those with T2D, compared with ND (Figure 2B, approximately two‐fold decrease in T1D compared with healthy individuals). In addition, and similarly to the CD4^+^ T cell compartment, the frequency of individuals in whom S‐specific CD8^+^ T cells could be detected was significantly lower in T1D compared with ND controls (22 out of 44 ND and 10 out of 44 T1D participants, *p* = 0.0142), while no differences were observed between T2D and ND (Figure 2C). Most S‐specific CD8^+^ T cells displayed a naïve or terminally differentiated T_EMRA_ phenotype, and there were no differences in the distribution of cells in naïve and memory subtypes among study groups (Figure S4).

Cytokine secretion patterns in participants with diabetes were clearly different from those of ND controls (Figure 2D). Both ND and diabetes groups contained similar frequencies of TNF‐ and granzyme B‐producing CD8^+^ T cells, with IFNγ and IL‐2 production being significantly increased in T1D and T2D compared with ND, respectively. In addition, while only small frequencies of S‐specific CD8^+^ T cells from the ND control group secreted other cytokines such as IL‐13, IL‐17A, or IL‐21, these were significantly increased in S‐specific CD8^+^ T cells from both T1D and T2D participants, suggesting an unfocused memory response to SARS‐CoV‐2 vaccination. No differences were observed in the production of IL‐10 among study groups (Figure 2D). Dimensionality reduction further confirmed that the global phenotype on a tSNE plot of S‐specific CD8^+^ T cells in participants with diabetes was different from that of ND (Figure 2E) and was more similar between T1D and T2D. Further corroborating the heterogeneity in the cytokine expression pattern, clustering analysis identified 14 different clusters of S‐specific CD8^+^ T cells (Figure 2F; Figure S5). Participants with diabetes displayed an altered distribution of cells among clusters compared with ND controls, with a specific enrichment of S‐specific CD8^+^ T cells in clusters 10, 12, and 13 and a significant decrease in the frequency of S‐specific CD8^+^ T cells in clusters 3 and 9 (Figure 2G; Figure S6). Clusters 10, 12, and 13 contained cells producing Th2‐ and Tfh‐related cytokines (IL‐13 in clusters 10, 11, and 12, IL‐21 in clusters 12 and 13) as well as IL‐17A (clusters 10 and 13) and IL‐10 (cluster 13). Cluster 3 was characterized by low levels of IL‐2 and IFNγ production and the absence of granzyme B and IL‐17A, while cluster 9 predominantly produced IL‐2. These data suggest that people with diabetes display an unfocused S‐specific CD8^+^ T cell memory response characterized by an increase in cells producing Th2‐ and Tfh‐related cytokines.

### 2.2 S‐Specific T Cells at Baseline and T Cell Memory Maintenance

**FIGURE 3:**
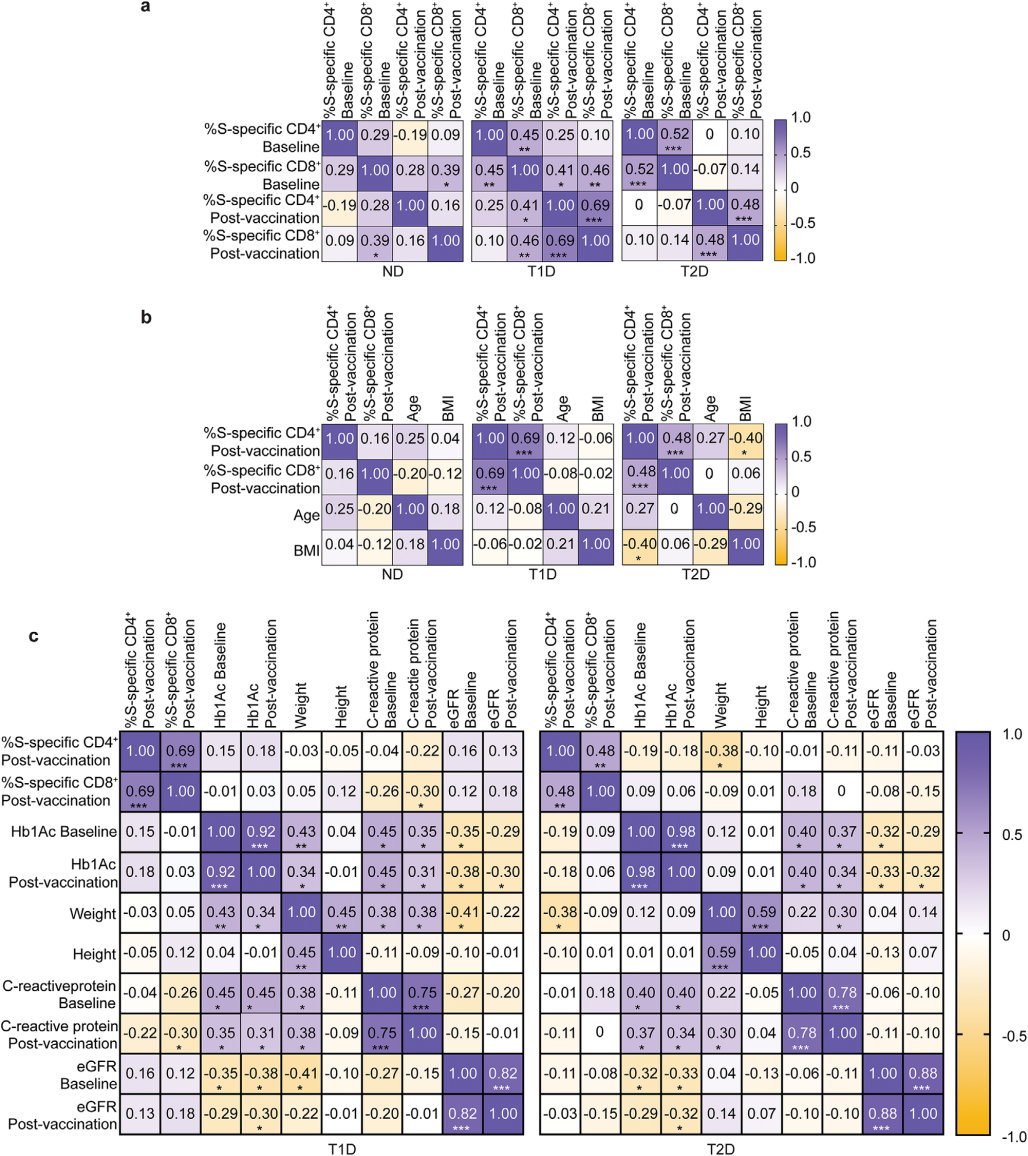
Cross‐reactivity and association with clinical parameters. **(A)** Correlation analysis of the frequency of S‐specific CD4^+^ and CD8^+^ T cells at baseline and after full vaccination protocol in ND controls (left), T1D (middle), and T2D (right) participants. **(B)** Correlation of frequency of S‐specific CD4^+^ and CD8^+^ T cells after full vaccination protocol with age and body mass index (BMI) in ND controls (left), T1D (middle), and T2D (right) patients. **(C)** Correlation of S‐specific CD4^+^ and CD8^+^ T cell frequency with clinical parameters at baseline and after full protocol of vaccination in T1D (left) and T2D (right) participants. Spearman's rank correlation coefficient (**A**, **B**, **C**). *p < 0.05; **p < 0.01; ***p < 0.005.

Some studies have reported the presence of pre‐existing T cells that recognize SARS‐CoV‐2 in uninfected naïve individuals, which have been suggested to enhance immune responses after SARS‐CoV‐2 infection and vaccination [14]. Therefore, we explored the association of S‐specific CD4^+^ and CD8^+^ T cells at baseline with the maintenance of memory to vaccination in ND controls, T1D, and T2D participants (Figure 3). Correlation analyses demonstrated that the percentage of S‐specific CD8^+^ T cells, but not of CD4^+^ T cells, at baseline positively correlated with the magnitude of CD8^+^ and of both CD8^+^ and CD4^+^ S‐specific T cell responses after vaccination in ND controls and T1D participants, respectively (Figure 3A). This correlation was not observed in T2D. Because there is a close association between obesity and T2D [15], and people with obesity experience an accelerated decline in vaccine‐specific B cell responses [16], we assessed whether vaccine‐specific T cell responses were correlated with obesity using body mass index (BMI) as a redout (Figure 3B). While no significant correlations were observed between S‐specific CD4^+^ or CD8^+^ T cell responses after vaccination and BMI or age in ND controls or the T1D group, the magnitude of S‐specific CD4^+^ T cell responses was negatively correlated with BMI in the T2D group. No correlation was observed between the frequency of CD4^+^ or CD8^+^ T cells at baseline and BMI (data not shown). We further examined whether the magnitude of the vaccine‐specific T cell response correlated with glycemia and other clinical parameters, including C‐reactive protein levels and estimated glomerular filtration rate (eGFR, Figure 3C), and only a negative correlation between the frequency of S‐specific CD8^+^ T cells after vaccination and C‐reactive protein levels was observed in participants with T1D.

These data suggest that the frequency of CD8^+^ T cells at baseline correlates with higher frequencies of both vaccine‐specific CD4^+^ and CD8^+^ T cells after vaccination in people with T1D and ND controls, but not in people with T2D. Moreover, obesity is negatively associated with the maintenance of vaccine‐specific CD4^+^ T cell memory in people with T2D.

### 2.3 Anti‐Inflammatory CD4^+^ T Cell Recall Responses to a Booster Dose of SARS‐CoV‐2 Vaccine in Diabetes

**FIGURE 4:**
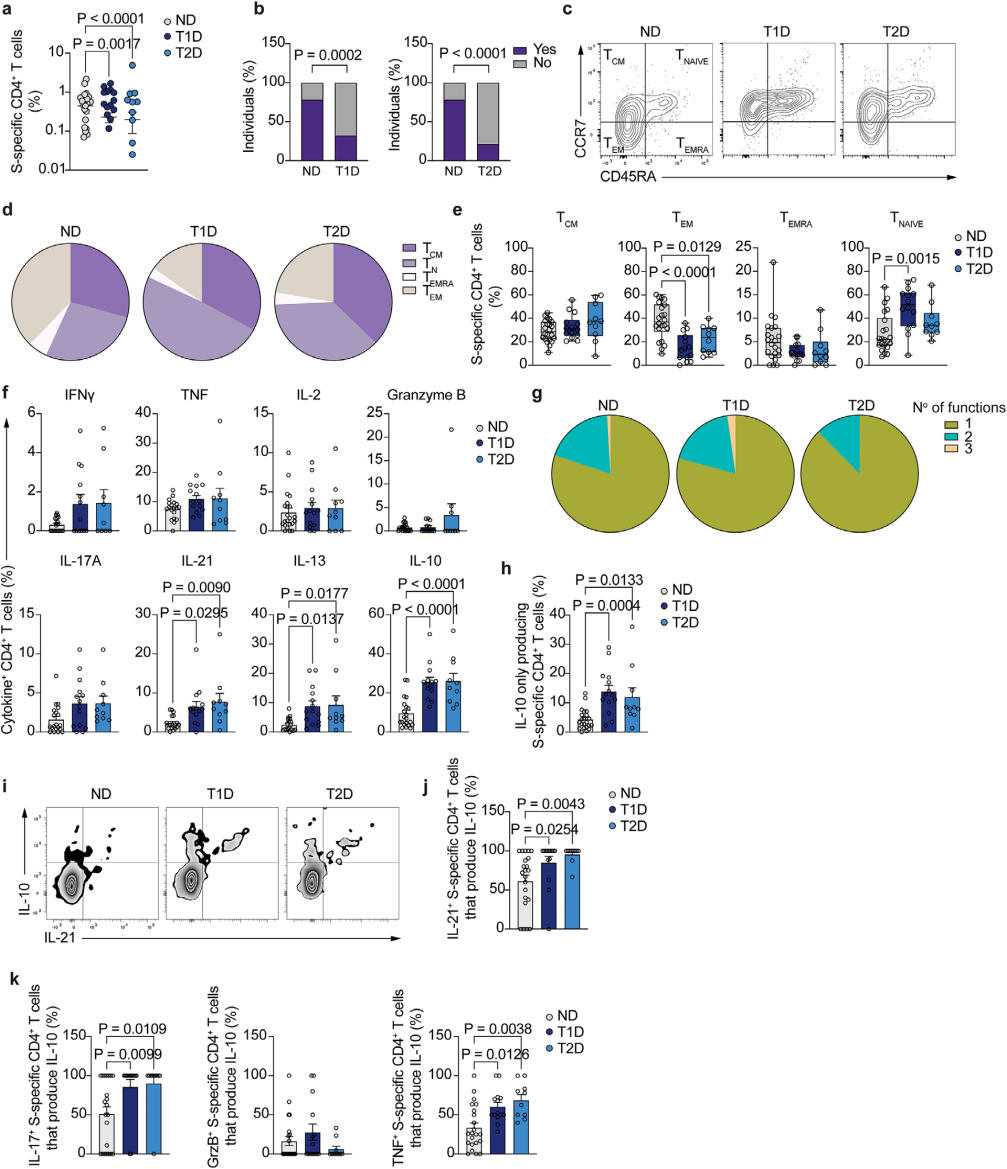
Vaccine‐specific CD4^+^ T cell recall responses. **(A)** Summary of frequency of S‐specific CD4^+^ T cells from total CD4^+^ T cells (*n =* 22 ND, *n =* 14 T1D, and *n =* 10 T2D). **(B)** Percentage of individuals in whom S‐specific CD4^+^ T cells were (purple) or not (grey) detected in healthy individuals (*n =* 29) and T1D patients (*n =* 44) (left), and healthy individuals (*n =* 29) and T2D patients (*n =* 46) (right). **(C)** Representative dot plot of the expression of CD45RA and CCR7 in S‐specific CD4^+^ T cells from healthy individuals (left), T1D (middle), and T2D (right) patients. **(D)** Percentage of S‐specific CD4^+^ T cells from healthy individuals (left, *n =* 22), T1D (middle, *n =* 14), and T2D (right, *n =* 10) with naïve, memory, central memory, or effector memory cells re‐expressing CD45RA phenotype. **(E)** Summary of S‐specific CD4^+^ T cells from healthy individuals (*n =* 22), T1D (*n =* 14), and T2D (*n =* 10) patients with naïve, memory, central memory, or effector memory S‐specific CD4^+^ T cells re‐expressing CD45RA phenotype. **(F)** Summary of the frequency of cytokine‐expressing S‐specific CD4^+^ T cells from healthy individuals (*n =* 19–22), T1D (*n =* 12–14), and T2D (*n =* 9–10). **(G)** Distribution of S‐specific CD4^+^ T cell polyfunctionality in healthy individuals (left), T1D (middle), and T2D (right) patients. **(H)** Percentage of S‐specific CD4^+^ T cells that only produce IL‐10. **(I)** IL‐10 and IL‐21 production by S‐specific CD4^+^ T cells. **(J)** Summary of the percentage of IL‐21‐producing, S‐specific CD4^+^ T cells that co‐produce IL‐10. **(K)** Summary of the percentage of IL‐17A‐ (left), granzyme B‐ (middle), and TNF‐producing (right) S‐specific CD4^+^ T cells that co‐produce IL‐10. One‐way ANOVA with Tukey's correction for multiple comparisons (**A**, **E**, **H**, **J**, **K)**, two‐tailed Fisher's exact test (**B**), Kruskal–Wallis test with correction for multiple comparisons (**F**).

We went on to examine the recall T cell response to a third booster dose of SARS‐CoV‐2 vaccine by characterizing the S‐specific T cell responses 2–4 weeks after vaccination. The frequency of S‐specific CD4^+^ T cells was significantly lower in both T1D and T2D participants when compared with that of ND controls (approximately 2‐ and 2.25‐fold decrease in T1D and T2D, respectively, Figure 4A). Additionally, the percentage of participants with T1D or T2D in which S‐specific CD4^+^ T cells were detected was significantly lower than that of ND (79% of ND compared with 33% of T1D and 22% of T2D patients, Figure 4B). Besides the decreased magnitude of the CD4^+^ T cell response, in those individuals where S‐specific CD4^+^ T cells were detected, the memory phenotype distribution was different among groups (Figure 4C), and while a large proportion of ND control T cells had an effector memory phenotype, T1D T cells were preferentially naïve (Figures 4D,E). In addition, both T1D and T2D participants displayed a decrease in the frequency of S‐specific CD4^+^ T cells with an effector memory phenotype (Figure 4E). These differences were not due to global changes in antigen experience distribution in total CD4^+^ T cells among groups (Figure S7).

The functionality of S‐specific CD4^+^ T cells was assessed by dissecting the pattern of cytokine production (Figure 4F). In those individuals in whom S‐specific CD4^+^ T cells were detected, no significant changes in the production of IFNγ, TNF, IL‐2, or granzyme B were observed among ND, T1D, and T2D participants. Polyfunctionality analysis using these four cytokines [13] (Figure 4G) demonstrated that the majority (>75%) of the vaccine‐specific CD4^+^ T cells in the 3 groups were producers of a single cytokine (either IFNγ, TNF, IL‐2, or granzyme B), and most of the remaining cells were bi‐functional. No significant differences in the percentage of single‐ or polyfunctional cells were found among ND controls, T1D, and T2D groups Figure S8).

We also measured the frequency of S‐specific CD4^+^ T cells producing IL‐17A, IL‐21, IL‐13, and IL‐10, and while no differences in IL‐17A production were observed, S‐specific CD4^+^ T cells from T1D and T2D displayed an increased frequency of IL‐21‐, IL‐13‐, and IL‐10‐producing cells, suggesting a Th2‐ and Tfh‐biased helper T cell response to vaccination (Figure 4f). We decided to further explore the increased secretion of IL‐10 by vaccine‐specific T cells to determine whether it originated from cells only producing IL‐10 or, on the contrary, from cells producing other helper T cell‐specific cytokines. The frequency of cells producing only IL‐10 and no other measured cytokine was significantly higher in both T1D and T2D participants compared with ND controls (Figure 4H). Moreover, the increase in IL‐21‐producing CD4^+^ T cells in participants with diabetes, which are important in providing B cell help and in enhancing the survival and function of cytotoxic CD8^+^ T cells upon vaccination or viral infection, was accompanied by a concomitant increase in IL‐10 production (Figure 4I). Thus, while only about 50% of IL‐21‐producing CD4^+^ T cells in HC were co‐expressing IL‐10, more than 85% and 95% S‐specific IL‐21‐producing CD4^+^ T cells were also producing IL‐10 in T1D and T2D, respectively (Figure 4J). Additionally, IL‐10 co‐production was not restricted to IL‐21‐producing cells, and most of IL‐17A‐secreting S‐specific CD4^+^ T cells were also expressing IL‐10 in T1D and T2D, compared with approximately 50% in ND control (Figure 4K). Similarly, a significantly increased percentage of TNF‐producing CD4^+^ T cells was co‐producing IL‐10 in T1D and T2D participants compared with ND (60% and 69% in T1D and T2D, respectively, compared with 33% in ND controls).

These data suggest that CD4^+^ T cell recall responses to SARS‐CoV‐2 vaccination are significantly impaired in people with diabetes at the time point analyzed (2–4 weeks after vaccination). Besides the significant decrease in participants in which detectable responses are identified at the time point analyzed, detectable S‐specific CD4^+^ T cells are characterized by an anti‐inflammatory, IL‐10‐mediated phenotype that includes not only the appearance of an IL‐10‐producing population, but also the acquisition of IL‐10 expression by most helper CD4^+^ T cells.

### 2.4 Anti‐Inflammatory CD8^+^ T Cell Recall Response to a Third Booster Dose of SARS‐CoV‐2 Vaccine in Diabetes

**FIGURE 5:**
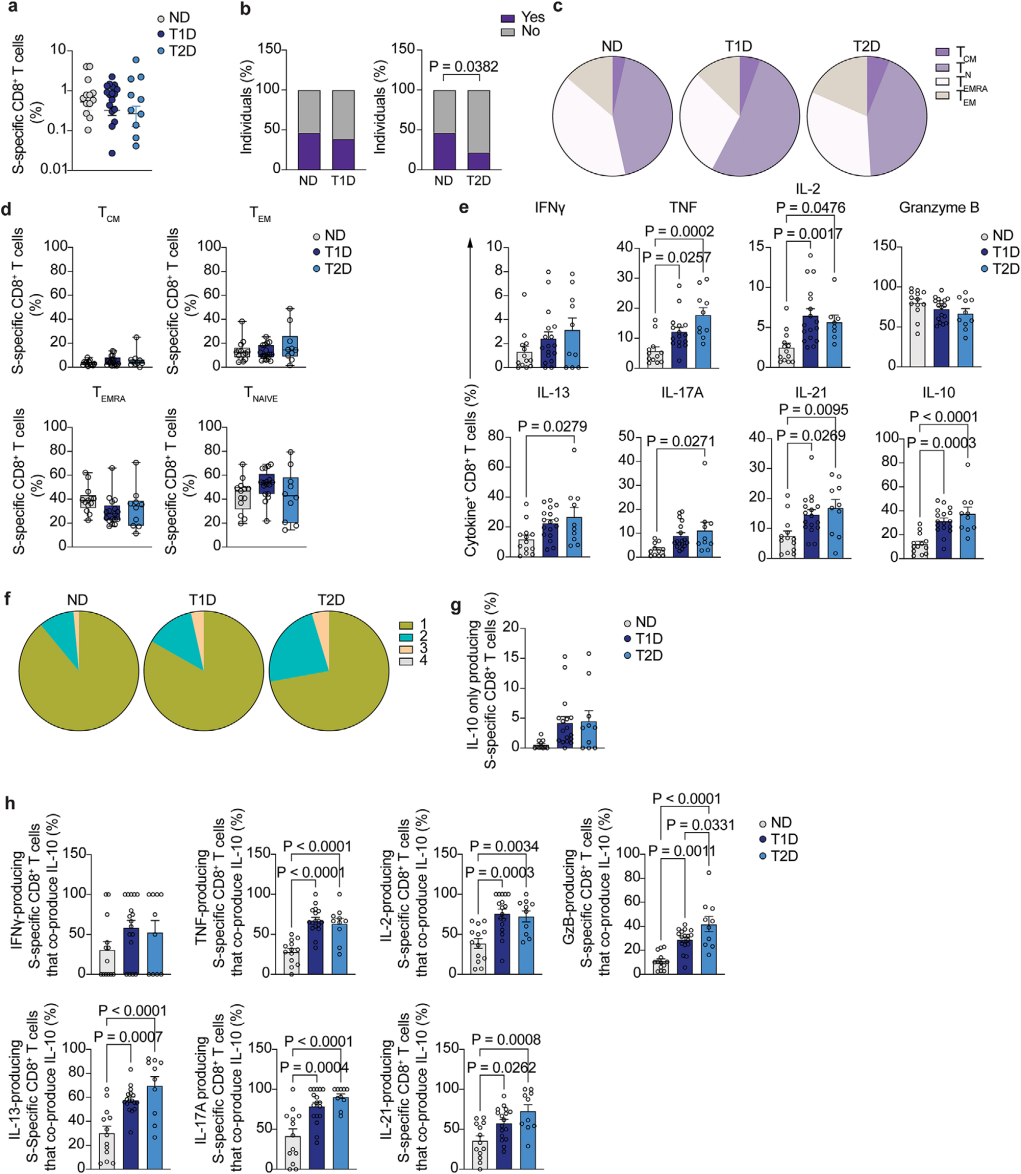
Vaccine‐specific CD8^+^ T cell recall responses. **(A)** Summary of frequency of S‐specific CD8^+^ T cells from total CD8^+^ T cells (*n =* 13 ND, *n =* 17 T1D, and *n =* 10 T2D). **(B)** Percentage of individuals in whom S‐specific CD8^+^ T cells were (purple) or not (grey) detected in healthy individuals (*n =* 29) and T1D patients (*n =* 44) (left), and healthy individuals (*n =* 29) and T2D patients (*n =* 46) (right). **(C)** Percentage of S‐specific CD8^+^ T cells from healthy individuals (left, *n =* 13), T1D (middle, *n =* 17), and T2D (right, *n =* 10) with naïve, memory, central memory, or effector memory cells re‐expressing CD45RA phenotype. **(D)** Summary of S‐specific CD8^+^ T cells from healthy individuals (*n =* 13), T1D (*n =* 17), and T2D (*n =* 10) patients with naïve, memory, central memory, or effector memory S‐specific CD8^+^ T cells re‐expressing CD45RA phenotype. **(E)** Summary of the frequency of cytokine‐expressing S‐specific CD8^+^ T cells from healthy individuals (*n =* 11–13), T1D (*n =* 16–17), and T2D (*n =* 8–10). **(F)** Distribution of S‐specific CD8^+^ T cell polyfunctionality in healthy individuals (left), T1D (middle), and T2D (right) patients. **g**. Summary of the percentage of S‐specific CD8^+^ T cells that only produce IL‐10. **(H)** Summary of the percentage of cytokine‐producing S‐specific CD8^+^ T cells that co‐produce IL‐10. One‐way ANOVA with Tukey's correction for multiple comparisons (**A**, **D)**, Two‐tailed Fisher's exact test (**B**), Kruskal–Wallis test with correction for multiple comparisons (**E**, **G**, **H**).

We subsequently analyzed the vaccine‐specific CD8^+^ T cell recall response in ND controls, T1D, and T2D participants (Figure 5). The frequency of S‐specific CD8^+^ T cells after the SARS‐CoV‐2 vaccine booster dose was similar among ND, T1D, and T2D participants, and no significant differences were observed in the percentage of individuals in whom S‐specific CD8^+^ T cells were detected between the ND and T1D groups (Figure 5A). However, a significant decrease in the percentage of individuals with T2D that had detectable vaccine‐specific CD8^+^ T cell responses compared with ND was observed (Figure 5B). The distribution of antigen experience of vaccine‐specific CD8^+^ T cells was not significantly different among ND controls and people with diabetes (Figure 5C), with similar proportions of S‐specific CD8^+^ T cells with a naïve and T_EMRA_ phenotype, and lower proportions of effector memory and central memory phenotypes (Figure 5D). This cellular distribution of antigen experience was not different from that of total CD8^+^ T cells from the three study groups, although the diabetes groups showed a modest increase in the frequency of total CD8^+^ T cells with a central memory phenotype (Figure S9). To assess functionality, the secretion of TNF, IFNγ, IL‐2, and granzyme B were measured as a readout for an efficient CD8^+^ T cell response to vaccination [13]. In addition, IL‐21, IL‐17A, IL‐13, and IL‐10 were assessed as readouts for cells with Tc21, Tc17, Tc2, and anti‐inflammatory phenotypes, respectively (Figure 5E). Participants with T1D and with T2D harbored an increased frequency of TNF‐ and IL‐2‐secreting S‐specific CD8^+^ T cells, while no differences were observed in the production of IFNγ or granzyme B compared with ND controls. We used these four cytokines to explore polyfunctionality of the CD8^+^ T cell recall response, and while most cells were expressing only one function, there was a significant increase in the percentage of S‐specific CD8^+^ T cells that produced a combination of 2 and 3 cytokines in T2D participants compared with ND (Figure 5F). S‐specific CD8^+^ T cells in T1D participants also displayed an increased frequency of cells co‐producing 3 cytokines, suggesting a significant increase in polyfunctionality in both T1D and T2D patients compared with ND (Figure S10).

However, the frequency of cells producing IFNγ, TNF, and IL‐2 was significantly lower than that of S‐specific CD8^+^ T cells producing other helper cytokines. Thus, increased expression of IL‐21 and IL‐10 was detected in people with T1D compared with ND (31% and 37% of IL‐10‐producing CD8^+^ T cells in T1D and T2D participants, respectively, compared with 12% in people without diabetes, Figure 5E). Similarly, S‐specific CD8^+^ T cells from participants with T2D produced significantly increased IL‐13, IL‐17A, IL‐21, and IL‐10.

The significantly increased production of IL‐10 by S‐specific CD8^+^ T cells in diabetes groups, potentially detrimental for an optimal vaccine‐specific T cell response, prompted us to examine its pattern of expression further, and we explored the possibility of a CD8^+^ T cell regulatory population arising in diabetes characterized by IL‐10 expression. However, no significant increase in the frequency of S‐specific CD8^+^ T cells secreting only IL‐10 (and not any other cytokine assessed) was observed in the diabetes groups compared with ND (Figure 5G). Instead, when we examined whether the secretion of IL‐10 was associated with any specific Tc population (identified by the expression of their canonical cytokines [17]), we found that a significant proportion of TNF‐, granzyme B‐ and IL‐2‐, IL‐13‐, IL‐17A‐, and IL‐21‐secreting cells were co‐producing IL‐10 in people with diabetes as compared with ND participants (Figure 5H), suggesting the acquisition of IL‐10 production capacity by most Tc types (Figure S11).

## 3 Discussion

In this work, we have dissected the cellular response to vaccination in individuals with T1D and T2D, focusing on memory maintenance, magnitude, and nature of the vaccine‐specific CD4^+^ and CD8^+^ T cell responses. Our results suggest an impairment in memory generation and/or maintenance in T1D compared with people without diabetes at the time points analyzed, both in the CD4^+^ and CD8^+^ T cell compartments, which is accompanied by a decreased frequency of vaccine‐specific T cells after the full vaccination protocol. We also identified significant alterations in the functionality of these cells, with an increased secretion of the Th2‐related cytokine IL‐13. Recall responses to a booster dose of SARS‐CoV‐2 vaccination were also impaired in both T1D and T2D, with a significant percentage of participants with diabetes failing to mount a S‐specific T cell response at the time point analyzed. Interestingly, in those participants with diabetes where a response was detected, it was dominated by the secretion of IL‐10 that was present in most cytokine‐producing cells.

Studies exploring the immune responses to SARS‐CoV‐2 vaccination in T2D have mostly measured vaccine‐specific antibody titers and show conflicting results, with some works suggesting a diminished antibody response after vaccination [7, 18] and other reports demonstrating similar antibody titers between diabetes participants and ND controls [19, 20]. The magnitude of vaccine‐specific T cell responses in our T2D cohort, however, resembled those found in people without diabetes, in line with other studies identifying similar T cell responses to SARS‐CoV‐2 vaccination in ND controls and people with T2D vaccinated with the BNT162b2 vaccine. Interestingly, humoral and cellular responses to whole inactivated SARS‐CoV‐2 vaccines are decreased in people with T2D [21], and vaccine type seems to differentially affect antibody responses in diabetes [22]. The impaired immunological response to SARS‐CoV‐2 vaccination and booster dose in people with diabetes does not seem to be specific to SARS‐CoV‐2, because similar results have been observed for the hepatitis B vaccine [6]. In our study, participants with T1D displayed most of the alterations in frequency and T cell memory maintenance after the full protocol of vaccination compared with T2D and ND controls. These data are in agreement with a recent study measuring T‐cell‐derived cytokines as a readout for T‐cell responses to SARS‐CoV‐2 vaccination [23] and an early study examining T‐cell responses to other vaccines in T1D [24]. Indeed, an impairment in the immune response following vaccination in patients with T1D has been previously suggested for other immunization strategies, including Influenza, rotavirus, and Influenza type B [24, 25], although underlying mechanisms remained elusive. In our study, people with T1D displayed a significantly lower frequency of CD4^+^ T cells after the full vaccination protocol and after the booster dose, and this was accompanied by a significant reduction in the percentage of people with T1D with S‐specific CD4^+^ T cell responses compared with ND. Several mechanisms could explain these results, including a defect in the maintenance of memory CD4^+^ T cells to vaccination in people with T1D, and a delayed T cell response to vaccination (Figures 4 and 5). Due to the limited number of time points available for analysis after the full course of vaccination or the recall response, our results can only suggest a delayed response to vaccination as the underlying mechanism for the small number of people with T1D and T2D who responded to the booster dose at the time point analyzed.

Systems biology and clinical studies on the response to SARS‐CoV‐2 vaccines in humans indicate that efficient vaccine‐specific CD4^+^ T cell responses are characterized by a Th1‐like profile and either low or absent levels of the Th2 cytokines IL‐4 and IL‐5 [26, 27]. The unfocused and tolerogenic recall response to SARS‐CoV‐2 vaccination, dominated by the acquisition of IL‐10 and IL‐13 production by vaccine‐specific T cells from people with diabetes, is in contrast with the focused, TNF‐, IL‐2‐ IFNγ‐producing T cell response observed by us and others [28] in people without diabetes, where vaccination barely induced any Th2‐ or Th17‐type cytokine.

Whilst the induction of IL‐13 has been associated with reduced ACE2 expression and viral entry [29, 30], and Th2 cytokines could potentially be beneficial in mucosal immunity [31], IL‐13 has also been suggested to be a predictor for COVID‐19 severity in humans [32] and mouse models [30], with IL‐13 blockade increasing the activity and polyfunctionality of CD8^+^ T cell responses in both vaccination settings [33, 34] and upon Influenza infection [35, 36]. The observation that IL‐10 production is acquired by vaccine‐specific CD4^+^ and CD8^+^ T cells with a variety of cytokine profiles and is not restricted to a specific T helper or Tc subpopulation suggests that, in diabetes, vaccination generates a generalized anti‐inflammatory response, potentially reducing the efficacy of the vaccine. However, the pleiotropic nature of IL‐10 indicates that, alongside immunosuppressive functions, IL‐10 can also be immunostimulatory, driving B cell differentiation into plasma cells [37] as well as enhancing and maintaining SARS‐CoV‐2‐specific tissue resident memory T cells in the nasal mucosa [38]. Additionally, elevated IL‐10 has been identified as a correlate for severe COVID‐19, with evidence suggesting that the timing of IL‐10 induction could dictate the efficacy of the immune response. Indeed, early induction of IL‐10 limits antigen‐specific CD4^+^ T cell expansion, function, and secondary recall responses during phagosomal infections [39], and IL‐10 receptor blockade delivered simultaneously with BCG vaccine stimulates immunity and sustains long‐term protection in mice [40]. Thus, further investigations are warranted to determine the balance between protective and tolerogenic functions of vaccine‐specific T cell‐derived IL‐10 and IL‐13 in people with diabetes and to unambiguously define the involvement of IL‐10 and IL‐13 in the maintenance and function of vaccine‐specific T cell responses, as well as their favorable or detrimental role in antiviral immunity upon infection in diabetes. Existing data indicate that expansion of Th1 CD4^+^ cells with the capacity to secrete IFNγ, TNF, and IL‐2 upon SARS‐CoV‐2 and other respiratory viral infections is associated with improved protection and viral control as opposed to an enhanced type 2 response [41, 42, 43, 44, 45]. Therefore, our data suggest that people with T1D, and to a lesser extent, people with T2D, mount an inefficient cellular response to vaccination at the time point analyzed.

Our results have implications for SARS‐CoV‐2 and potentially other vaccination regimens for people with diabetes and warrant further investigations to pinpoint the mechanisms underlying the defects observed, the involvement of other factors, including hyperglycemia and chronic inflammation, and broader studies focused on vaccine effectiveness in diabetes populations.

### 3.1 Data Limitations and Perspectives

This study has some technical limitations, including the lack of longitudinal samples after the booster dose to determine whether the differences in vaccine‐specific T cell frequencies in diabetes groups are due to a delayed or simply a lack of response, and the small cell numbers used for the clustering analyses in Figures 1 and 2 (Table S2). Moreover, while our antigen experience measurements defined naïve T cells as CD45RA^+^CCR7^+^, this population could also include a small proportion of memory cells with a stem cell‐like phenotype [46]. Finally, while the majority of participants received mRNA‐based vaccines, a small percentage received the ChAdOx1 vaccine, which could act as a confounding factor in the analysis of the results [47]. However, no differences in the frequency of S‐specific CD4^+^ and CD8^+^ T cells were observed when participants were compared based on the vaccine type received (data not shown).

## 4 Materials and Methods

### 4.1 Participants and Clinical Data Collection

The cohorts analyzed in this study consisted of individuals aged 18 years or older, either without known diabetes or with a diagnosis of T1D or T2D. Participants with diabetes were recruited to the COVAC‐DM study, a prospective, multicenter, cohort study aimed at examining the immune response to COVID‐19 vaccination in people with type 1 and 2 diabetes mellitus and a glycated hemoglobin level ≤58 mmol/mol (7.5%) or >58 mmol/mol, respectively (EudraCT2021‐001459‐15) [19, 20]. Participants were administered the full 2‐dose course and a third “booster” dose of BTN162b2 (Pfizer/BioNTech), ChAdOx1‐S (AstraZeneca) (only for the first 2 vaccination doses and only used in 8 participants [5.3% of the entire study]), or mRNA‐1273 (Moderna) vaccines between April 2021 and March 2022. Blood samples were collected at baseline (60‐2 days before their first vaccination), 4–16 weeks after the second dose, and 2–4 weeks after the third dose, after informed consent was obtained. None of the T1D and T2D had had COVID‐19 either before or during the length of the study, and both diabetes groups were matched for HbA1c. The control group without diabetes consisted of individuals from the CoVVac (EudraCT: 2021‐001040‐10) study and additional participants from the COVIDITY study, a prospective observational serial sampling study of whole blood that recruited people with suspected or confirmed SARS‐CoV‐2 infection who were followed for 18 months. Twelve healthy individuals who were included in the data analysis in Figures 1 and 2 were participants from the COVIDITY study and had COVID‐19 at least 12 months before COVID‐19 vaccination (Table S1).

Participants from both cohorts were recruited at the University Hospital Graz, either from established cohorts (Graz Diabetes Registry for Biomarker Research), the outpatient clinic for diabetes, lipid disorders, and metabolism, or via advertisements. Main exclusion criteria for the COVAC‐DM trial were: active malignancy (excluding intraepithelial neoplasia of the prostate gland and the gastrointestinal tract and basalioma), pregnancy, acute inflammatory disease, immunosuppressant therapy, alcohol abuse (more than 15 standard drinks a week), or any contraindication to the vaccine, as well as a previous episode of COVID‐19.

The CoVVac trial included healthy controls without known diabetes and excluded people with the presence of disease or therapy potentially interfering with the response to vaccination. All participants provided informed consent at the time of recruitment.

### 4.2 PBMC Isolation and Storage

Blood samples (40 mL) were collected using BD Vacutainer CPT (Becton Dickinson, NJ, USA) tubes to separate mononuclear cells. Peripheral blood mononucleated cells were frozen in DMSO‐based media and stored in liquid nitrogen until further analysis.

### 4.3 Identification of S‐Specific T Cells

PBMC were thawed in a prewarmed water bath at 37°C and immediately transferred to a 15 mL conical tube, where 5 mL of prewarmed (37°C) cell culture media supplemented with 50 U/mL of Pierce Universal Nuclease (Thermo Fisher Scientific) or Benzonase (Merck) was added. Cell culture media used was RPMI 1640 (GIBCO) supplemented with 2 mM L‐Glutamine, 5% human serum AB (Sigma‐Aldrich), and 100 µg/mL penicillin and streptomycin (GIBCO). Samples were subsequently centrifuged for 10 min at 300×*g* and resuspended in warm cell culture media at 2 × 10^7^ cells/mL. PBMCs were rested for 2 h at 37°C, 5% CO_2,_ before ex vivo stimulation. One to two million PBMCs were incubated with 1 µg/mL anti‐CD40 (Miltenyi Biotec) and 1 µg/mL anti‐CD28 (Miltenyi Biotec) for 20 min at 37°C. Cells were then stimulated with either PepTivator SARS‐CoV‐2 complete S protein (Wuhan wild‐type, Miltenyi Biotec) or vehicle for 16 h. PMA (50 ng/mL) and ionomycin (250 ng/mL) stimulation was carried out in some samples as a positive control for stimulation, following the same protocol. For intracellular stainings, GolgiStop (BD Biosciences) and GolgiPlug (BD Biosciences) were added to the cultures after 12 h of stimulation for 4 h

### 4.4 Cellular Staining for Flow Cytometry

Cells were stained with LIVE/DEAD Fixable Blue Dead Cell Dye (Thermo Fisher Scientific) according to the manufacturer's specifications. A FcR receptor blocking step with FcR Blocking Reagent Human (Miltenyi Biotec) was carried out before cell surface antibody staining, which included antibodies to CD3 (BV785, clone UCHT1), CD4 (PerCP, clone RPA‐T4), CD154 (PE, clone 24–31), CD137 (BV711, clone 4B4‐1), CD69 (Alexa Fluor 700, clone FN50), CD45RA (BV650, clone HI100), and CCR7 (APC‐Cy7, clone G043H7), all from Biolegend. The cells were subsequently fixed using the FoxP3/Transcription Factor Staining Buffer kit (Thermo Fisher Scientific) following the manufacturer's specifications. Cells were stained with antibodies to intracellular targets, including anti‐human IL‐13 (BV421, clone JES10‐5A2), IL‐17A (BV510, clone BL168), granzyme B (FITC, clone QA16A02), IL‐2 (PeCP‐Cy5.5, clone MQ1‐17H12), TNF (PE Dazzle 594, clone Mab11), IL‐10 (PE‐Cy7, clone JES3‐9D7), from Biolegend and IL‐21 (eFluor 660, clone eBio3A3‐N2) and IFNγ (BV605, clone XMG1.2), from Thermo Fisher Scientific, washed and resuspended in 220 µL of PBS.

The samples were run on a Fortessa instrument (BD Biosciences) and analyzed using FlowJo v10.0 (BD Biosciences). Frequency of cytokine‐producing, S‐specific CD4^+^ and CD8^+^ T cells was calculated from the CD69^+^CD154^+^ and the CD69^+^CD137^+^ gates, respectively.

### 4.5 Dimensionality Reduction and Cluster Analysis

Dimensionality reduction and tSNE plots were obtained as previously described [48, 49]. Briefly, S‐specific CD4^+^ T cells (or S‐specific CD8^+^ T cells) from healthy individuals and participants with T1D and T2D were concatenated. The concatenated sample was used to calculate tSNE axes using 1500 iterations, perplexity of 30, and the default learning rate. In order to obtain cell clusters, the Phenograph plugin in FlowJo was used, with all compensated parameters.

### 4.6 Statistical Analysis

The frequency of S‐specific CD4^+^ and CD8^+^ T cells was calculated by subtracting the value of the vehicle‐stimulated cells for each study participant. Only those participants in whom S‐specific CD4^+^ or CD8^+^ T cells were detected after subtraction of background were included in further analyses, that is, cytokine analysis, antigen experience. Data were analyzed using GraphPad Prism version 10.0. Normal distribution of the data was tested using the D'Agostino and Pearson and Anderson‐Darling normality tests or the Shapiro–Wilk test for those datasets with a small number of data points. Normally distributed data by at least one of the two tests were analyzed using one‐ or two‐way ANOVA when comparing more than two groups of one or two independent variables, respectively. A two‐tailed *t*‐test was used to compare two groups. For non‐Gaussian distributed data, the Mann–Whitney *U* test or the Kruskal–Wallis test with correction for multiple comparisons was used to compare two or more groups, respectively. Fisher's exact test was used to determine the presence of a nonrandom association between two categorical variables, and Spearman's rank correlation coefficient was used to determine relationships between two variables. Data are represented as mean ± SEM. Where the data are presented as box and whiskers, the boxes extend from the 25th to the 75th percentile, and the whiskers are drawn down to the minimum and up to the maximum values. Horizontal lines within the boxes denote the average. *p*‐values <0.05 were considered statistically significant.

## Author Contributions

Emma M. Jones performed experiments, analyzed data, and wrote the manuscript. Caren Sourij, Martin Stradner, Peter Schlenke, Nazanin Sereban, Othmar Moser, and Harald Sourij designed and performed the COVAC‐DM and CoVVac trials. Harald Sourij, Martin Stradner, and Caren Sourij obtained funding for the clinical trials. Charlotte‐Eve Short and Graham P. Taylor designed, supervised, and managed the COVIDITY observational study. Rachael Quinlan recruited and processed samples from the COVIDITY study. Benjamin H. L. Harris and Michael Fertleman recruited participants for the COVIDITY study. Nick Oliver designed the study and wrote the manuscript. Harald Sourij designed the study and obtained funding. Margarita Dominguez‐Villar designed the study, analyzed data, wrote the manuscript, and obtained funding. All authors revised and contributed to the editing of the manuscript.

## Conflicts of Interest

The authors declare no conflicts of interest.

## Ethical Approval Statement for Human Studies

Ethical approvals were obtained from the ethics committee of the Medical University of Graz (33‐366 ex 20/21) and the South‐Central Oxford C Research Ethics Committee (IRAS ID 15/sc/0089).

## Supporting information




**Supporting File 1**: eji70112‐sup‐0001‐SuppMat.pdf

## Data Availability

The raw numbers for charts and graphs are available in the source data file whenever possible. The data that support the findings of this study are available from the corresponding author upon reasonable request.

## References

[1] J. Casqueiro , J. Casqueiro , and C. Alves , “Infections in Patients With Diabetes Mellitus: A Review of Pathogenesis,” Indian Journal of Endocrinology Metabolism 16, no. Suppl 1 (2012): S27–S36. 22701840 10.4103/2230-8210.94253 PMC3354930

[2] L. M. Muller , K. J. Gorter , E. Hak , et al., “Increased Risk of Common Infections in Patients With Type 1 and Type 2 Diabetes Mellitus,” Clinical Infectious Diseases 41 (2005): 281–288, 10.1086/431587. 16007521

[3] G. Dos Santos , H. Tahrat , and R. Bekkat‐Berkani , “Immunogenicity, Safety, and Effectiveness of Seasonal Influenza Vaccination in Patients With Diabetes Mellitus: A Systematic Review,” Human Vaccines & Immunotherapeutics 14 (2018): 1853–1866, 10.1080/21645515.2018.1446719. 29517396 PMC6149986

[4] T. Verstraeten , M. A. Fletcher , J. A. Suaya , et al., “Diabetes Mellitus as a Vaccine‐Effect Modifier: A Review,” Expert Review of Vaccines 19 (2020): 445–453, 10.1080/14760584.2020.1760098. 32516066

[5] R. Hopkins , K. G. Young , N. J. Thomas , et al., “Risk Factor Associations for Severe COVID‐19, Influenza and Pneumonia in People With Diabetes to Inform Future Pandemic Preparations: UK Population‐Based Cohort Study,” BMJ Open 14 (2024): e078135, 10.1136/bmjopen-2023-078135. PMC10831438 38296292

[6] S. F. Schillie , P. R. Spradling , and T. V. Murphy , “Immune Response of hepatitis B Vaccine Among Persons With Diabetes: A Systematic Review of the Literature,” Diabetes Care 35 (2012): 2690–2697, 10.2337/dc12-0312. 23173138 PMC3507602

[7] R. Marfella , N. D'Onofrio , C. Sardu , et al., “Does Poor Glycaemic Control Affect the Immunogenicity of the COVID‐19 Vaccination in Patients With Type 2 Diabetes: The CAVEAT Study,” Diabetes, Obesity & Metabolism 24 (2022): 160–165, 10.1111/dom.14547. PMC8653151 34494705

[8] F. Aberer , O. Moser , F. Aziz , et al., “Impact of COVID‐19 Vaccination on Glycemia in Individuals with Type 1 and Type 2 Diabetes: Substudy of the COVAC‐DM Study,” Diabetes Care 45 (2022): e24–e26, 10.2337/dc21-1563. 34848490

[9] A. Boyd , J. R. Almeida , P. A. Darrah , et al., “Pathogen‐Specific T Cell Polyfunctionality Is a Correlate of T Cell Efficacy and Immune Protection,” PLoS ONE 10 (2015): e0128714, 10.1371/journal.pone.0128714. 26046523 PMC4457486

[10] T. Lindenstrom , E. M. Agger , K. S. Korsholm , et al., “Tuberculosis Subunit Vaccination Provides Long‐Term Protective Immunity Characterized by Multifunctional CD4 Memory T Cells,” Journal of Immunology 182 (2009): 8047–8055, 10.4049/jimmunol.0801592. 19494330

[11] M. L. Precopio , M. R. Betts , J. Parrino , et al., “Immunization With vaccinia Virus Induces Polyfunctional and Phenotypically Distinctive CD8(+) T Cell Responses,” Journal of Experimental Medicine 204 (2007): 1405–1416, 10.1084/jem.20062363. 17535971 PMC2118607

[12] P. A. Darrah , D. T. Patel , P. M. De Luca , et al., “Multifunctional TH1 Cells Define a Correlate of Vaccine‐Mediated Protection Against Leishmania Major,” Nature Medicine 13 (2007): 843–850, 10.1038/nm1592. 17558415

[13] R. A. Seder , P. A. Darrah , and M. Roederer , “T‐cell Quality in Memory and Protection: Implications for Vaccine Design,” Nature Reviews Immunology 8 (2008): 247–258, 10.1038/nri2274. 18323851

[14] L. Loyal , J. Braun , L. Henze , et al., “Cross‐reactive CD4(+) T Cells Enhance SARS‐CoV‐2 Immune Responses Upon Infection and Vaccination,” Science 374 (2021): eabh1823, 10.1126/science.abh1823. 34465633 PMC10026850

[15] A. Abdullah , A. Peeters , M. de Courten , and J. Stoelwinder , “The Magnitude of Association Between Overweight and Obesity and the Risk of Diabetes: A Meta‐Analysis of Prospective Cohort Studies,” Diabetes Research and Clinical Practice 89 (2010): 309–319, 10.1016/j.diabres.2010.04.012. 20493574

[16] A. A. van der Klaauw , E. C. Horner , P. Pereyra‐Gerber , et al., “Accelerated Waning of the Humoral Response to COVID‐19 Vaccines in Obesity,” Nature Medicine 29 (2023): 1146–1154, 10.1038/s41591-023-02343-2. PMC10202802 37169862

[17] C. H. Koh , S. Lee , M. Kwak , B. S. Kim , and Y. Chung , “CD8 T‐cell Subsets: Heterogeneity, Functions, and Therapeutic Potential,” Experimental & Molecular Medicine 55 (2023): 2287–2299, 10.1038/s12276-023-01105-x. 37907738 PMC10689838

[18] Y. F. He , J. Ouyang , X. D. Hu , et al., “Correlation Between COVID‐19 Vaccination and Diabetes Mellitus: A Systematic Review,” World J Diabetes 14 (2023): 892–918, 10.4239/wjd.v14.i6.892. 37383586 PMC10294060

[19] C. Sourij , F. Aziz , H. Kojzar , et al., “Severe Acute Respiratory Syndrome Coronavirus 2 Spike Antibody Level Decline Is More Pronounced After the Second Vaccination, but Response to the Third Vaccination Is Similar in People With Type 1 and Type 2 Diabetes Compared With Healthy Controls: The Prospective COVAC‐DM Cohort Study,” Diabetes, Obesity & Metabolism 25 (2023): 314–318. 10.1111/dom.14855 PMC9538806 36057945

[20] C. Sourij , N. J. Tripolt , F. Aziz , et al., “Humoral Immune Response to COVID‐19 Vaccination in Diabetes Is Age‐Dependent but Independent of Type of Diabetes and Glycaemic Control: The Prospective COVAC‐DM Cohort Study,” Diabetes, Obesity & Metabolism 24 (2022): 849–858, 10.1111/dom.14643. PMC9303917 34984802

[21] C. H. Lee , V. Gray , J. M. N. Teo , et al., “Comparing the B and T Cell‐Mediated Immune Responses in Patients With Type 2 Diabetes Receiving mRNA or Inactivated COVID‐19 Vaccines,” Frontiers in immunology 13 (2022): 1018393, 10.3389/fimmu.2022.1018393. 36304475 PMC9592994

[22] F. Xiang , B. Long , J. He , et al., “Impaired Antibody Responses Were Observed in Patients With Type 2 Diabetes Mellitus After Receiving the Inactivated COVID‐19 Vaccines,” Virology journal 20 (2023): 22, 10.1186/s12985-023-01983-7. 36750902 PMC9902824

[23] F. D'Addio , G. Sabiu , V. Usuelli , et al., “Immunogenicity and Safety of SARS‐CoV‐2 mRNA Vaccines in a Cohort of Patients with Type 1 Diabetes,” Diabetes 71 (2022): 1800–1806, 10.2337/db22-0053. 35551366

[24] N. Eibl , M. Spatz , G. F. Fischer , et al., “Impaired Primary Immune Response in Type‐1 Diabetes: Results From a Controlled Vaccination Study,” Clinical Immunology 103 (2002): 249–259, 10.1006/clim.2002.5220. 12173299

[25] C. Remschmidt , O. Wichmann , and T. Harder , “Vaccines for the Prevention of Seasonal Influenza in Patients With Diabetes: Systematic Review and Meta‐Analysis,” BMC Medicine [Electronic Resource] 13 (2015): 53, 10.1186/s12916-015-0295-6. 25857236 PMC4373029

[26] P. S. Arunachalam , M. K. D. Scott , T. Hagan , et al., “Systems Vaccinology of the BNT162b2 mRNA Vaccine in Humans,” Nature 596 (2021): 410–416, 10.1038/s41586-021-03791-x. 34252919 PMC8761119

[27] U. Sahin , A. Muik , E. Derhovanessian , et al., “COVID‐19 Vaccine BNT162b1 Elicits Human Antibody and T(H)1 T Cell Responses,” Nature 586 (2020): 594–599, 10.1038/s41586-020-2814-7. 32998157

[28] L. Li , M. Muftuoglu , S. Liang , et al., “In‐Depth Analysis of SARS‐CoV‐2‐Specific T Cells Reveals Diverse Differentiation Hierarchies in Vaccinated Individuals,” JCI Insight 7 (2022), 10.1172/jci.insight.156559. PMC9057595 35230977

[29] H. E. Noh and M. S. Rha , “Mucosal Immunity Against SARS‐CoV‐2 in the Respiratory Tract,” Pathogens 13 (2024): 113, 10.3390/pathogens13020113. 38392851 PMC10892713

[30] S. Ghimire , B. Xue , K. Li , et al., “IL‐13 Decreases Susceptibility to Airway Epithelial SARS‐CoV‐2 Infection but Increases Disease Severity in Vivo via Eicosanoid Signalling,” EBioMedicine 120 (2025): 105920, 10.1016/j.ebiom.2025.105920. 40957220 PMC12466150

[31] M. F. Neurath , S. Finotto , and L. H. Glimcher , “The Role of Th1/Th2 Polarization in Mucosal Immunity,” Nature Medicine 8 (2002): 567–573, 10.1038/nm0602-567. 12042806

[32] A. N. Donlan , T. E. Sutherland , C. Marie , et al., “IL‐13 Is a Driver of COVID‐19 Severity,” JCI Insight 6 (2021): e150107. 34185704 10.1172/jci.insight.150107 PMC8410056

[33] L. P. Deimel , Z. Li , and C. Ranasinghe , “Interleukin‐13 as a Target to Alleviate Severe Coronavirus Disease 2019 and Restore Lung Homeostasis,” J Clin Transl Res 7 (2021): 116–120. 34027204 PMC8132187

[34] C. Ranasinghe and I. A. Ramshaw , “Immunisation Route‐Dependent Expression of IL‐4/IL‐13 Can Modulate HIV‐Specific CD8(+) CTL Avidity,” European Journal of Immunology 39 (2009): 1819–1830, 10.1002/eji.200838995. 19582753

[35] N. L. La Gruta , P. C. Doherty , and S. J. Turner , “A Correlation Between Function and Selected Measures of T Cell Avidity in Influenza Virus‐Specific CD8+ T Cell Responses,” European Journal of Immunology 36 (2006): 2951–2959. 17072910 10.1002/eji.200636390

[36] D. K. Wijesundara , R. J. Jackson , D. C. Tscharke , and C. Ranasinghe , “IL‐4 and IL‐13 Mediated Down‐Regulation of CD8 Expression Levels Can Dampen Anti‐Viral CD8(+) T Cell Avidity Following HIV‐1 Recombinant Pox Viral Vaccination,” Vaccine 31 (2013): 4548–4555, 10.1016/j.vaccine.2013.07.062. 23933364

[37] V. Carlini , D. M. Noonan , E. Abdalalem , et al., “The Multifaceted Nature of IL‐10: Regulation, Role in Immunological Homeostasis and Its Relevance to Cancer, COVID‐19 and Post‐COVID Conditions,” Frontiers in Immunology 14 (2023): 1161067, 10.3389/fimmu.2023.1161067. 37359549 PMC10287165

[38] C. E. Nelson , T. W. Foreman , E. R. Fukutani , et al., “IL‐10 Suppresses T Cell Expansion While Promoting Tissue‐Resident Memory Cell Formation During SARS‐CoV‐2 Infection in rhesus Macaques,” Plos Pathogens 20 (2024): e1012339, 10.1371/journal.ppat.1012339. 38950078 PMC11244803

[39] A. K. Singh and N. R. Thirumalapura , “Early Induction of Interleukin‐10 Limits Antigen‐Specific CD4(+) T Cell Expansion, Function, and Secondary Recall Responses During Persistent Phagosomal Infection,” Infection and Immunity 82 (2014): 4092–4103, 10.1128/IAI.02101-14. 25024370 PMC4187882

[40] V. Dwivedi , S. Gautam , C. A. Headley , T. Piergallini , J. B. Torrelles , and J. Turner , “IL‐10 Receptor Blockade Delivered Simultaneously With Bacillus Calmette‐Guerin Vaccination Sustains Long‐Term Protection Against Mycobacterium Tuberculosis Infection in Mice,” Journal of Immunology 208 (2022): 1406–1416, 10.4049/jimmunol.2100900. PMC11075079 35181640

[41] M. Han , K. Ma , X. Wang , et al., “Immunological Characteristics in Type 2 Diabetes Mellitus among COVID‐19 Patients,” Front Endocrinol (Lausanne) 12 (2021): 596518, 10.3389/fendo.2021.596518. 33776910 PMC7992040

[42] P. Moss , “The T Cell Immune Response Against SARS‐CoV‐2,” Nature Immunology 23 (2022): 186–193, 10.1038/s41590-021-01122-w. 35105982

[43] S. Notarbartolo , V. Ranzani , A. Bandera , et al., “Integrated Longitudinal Immunophenotypic, Transcriptional and Repertoire Analyses Delineate Immune Responses in COVID‐19 Patients,” Science Immunology 6 (2021), 10.1126/sciimmunol.abg5021. 34376481

[44] C. Rydyznski Moderbacher , S. I. Ramirez , J. M. Dan , et al., “Antigen‐Specific Adaptive Immunity to SARS‐CoV‐2 in Acute COVID‐19 and Associations With Age and Disease Severity,” Cell 183 (2020): 996–1012 e1019. 33010815 10.1016/j.cell.2020.09.038 PMC7494270

[45] A. Cerwenka , T. M. Morgan , A. G. Harmsen , and R. W. Dutton , “Migration Kinetics and Final Destination of Type 1 and Type 2 CD8 Effector Cells Predict Protection Against Pulmonary Virus Infection,” Journal of Experimental Medicine 189 (1999): 423–434, 10.1084/jem.189.2.423. 9892624 PMC2192982

[46] L. Gattinoni , E. Lugli , Y. Ji , et al., “A Human Memory T Cell Subset With Stem Cell‐Like Properties,” Nature Medicine 17 (2011): 1290–1297, 10.1038/nm.2446. PMC3192229 21926977

[47] Z. Kondera‐Anasz , E. J. Morawiec , R. Duszkiewicz , F. Hajdrowski , and A. Wiczkowski , “Comparison of SARS‐CoV‐2 Immune Responses Following Vaccination With Comirnaty (Pfizer) and Vaxzevria (AstraZeneca) in Healthy Individuals With or Without Prior SARS‐CoV‐2 Infection,” Frontiers in Immunology 16 (2025): 1612288, 10.3389/fimmu.2025.1612288. 40746556 PMC12310665

[48] A. K. Maher , A. Aristodemou , N. Giang , et al., “HTLV‐1 Induces an Inflammatory CD4+CD8+ T Cell Population in HTLV‐1‐Associated Myelopathy,” JCI Insight 9 (2024), 10.1172/jci.insight.173738. PMC10906466 38193535

[49] A. K. Maher , K. L. Burnham , E. M. Jones , et al., “Transcriptional Reprogramming From Innate Immune Functions to a Pro‐Thrombotic Signature by Monocytes in COVID‐19,” Nature Communications 13 (2022): 7947, 10.1038/s41467-022-35638-y. PMC9791976 36572683

