# Supplementary material for: IL‐10‐ and IL‐13‐Biased T Cell Responses to SARS‐CoV‐2 Vaccination in Diabetes
Source: Eur J Immunol. 2025 Dec 9;55(12):e70112. doi: 10.1002/eji.70112 (PMC12690275; doi:10.1002/eji.70112)
Supplement: Supplementary file 1 — Supporting File 1: eji70112‐sup‐0001‐SuppMat.pdf [file EJI-55-e70112-s001.pdf]

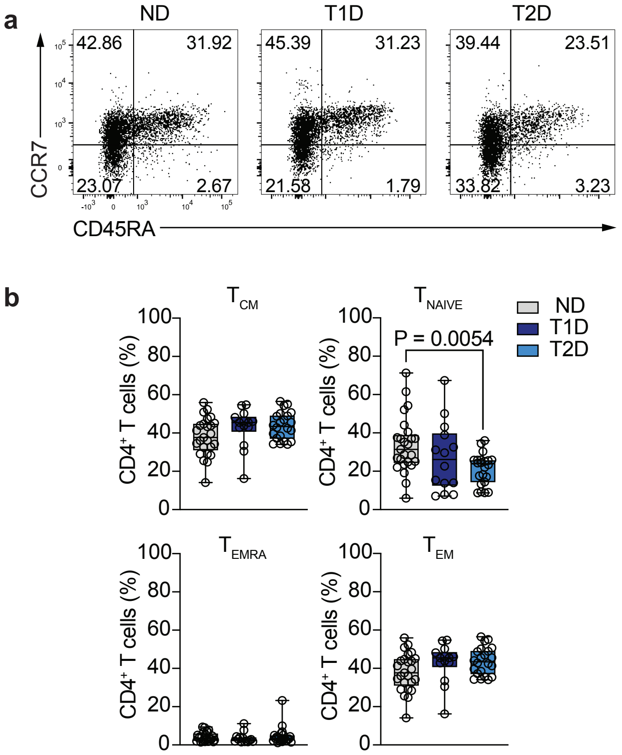


**Supplementary Figure 1. Total CD4$^+$ T cell antigen experience**. **a**. Representative dot plot of the expression of CD45RA and CCR7 in ND controls (left), T1D (middle) and T2D (right) participants. **b**. Percentage of CD4$^+$ T cells from ND (left), T1D (middle) and T2D (right) individuals with a naïve, memory, central memory or effector memory cells re-expressing CD45RA phenotype.

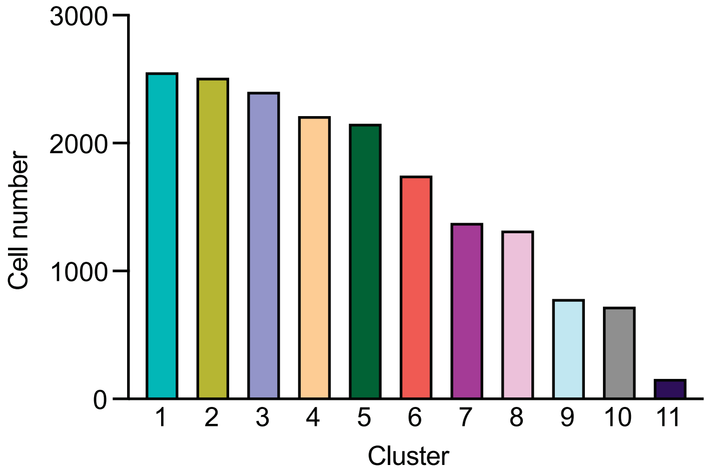


**Supplementary Figure 2. Cell numbers in S-specific CD4+ T cell cluster analysis.**

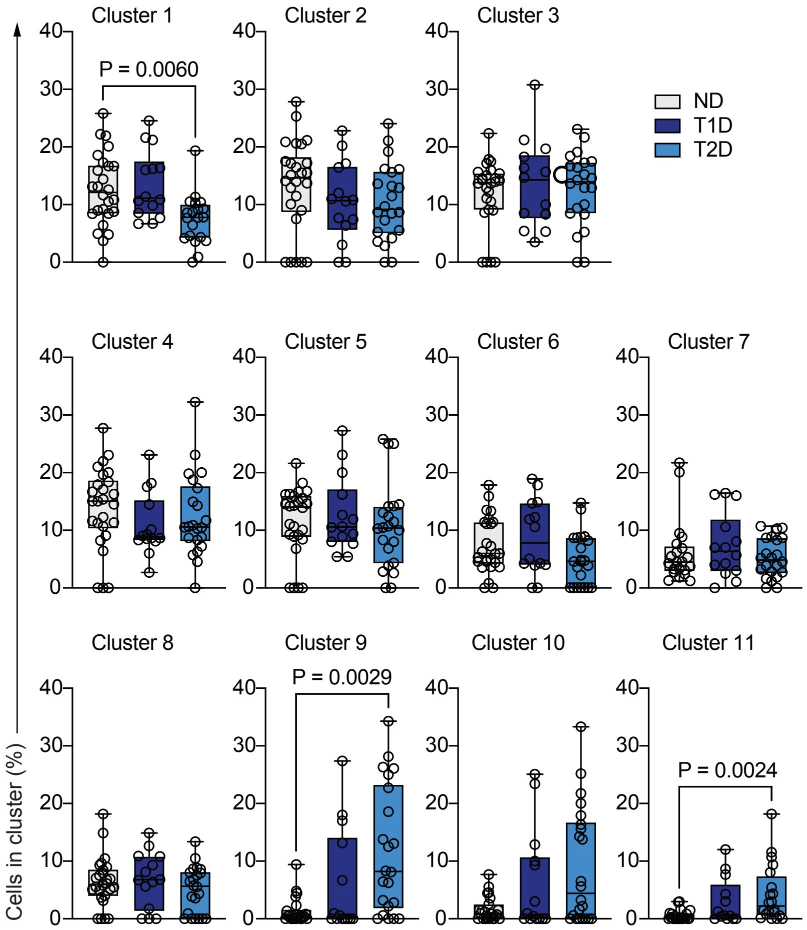


**Supplementary Figure 3. S-specific CD4⁺ T cell cluster distribution.** Percentage of S-specific CD4⁺ T cells in each of the identified clusters. One-way ANOVA with Tukey's correction for multiple comparisons.

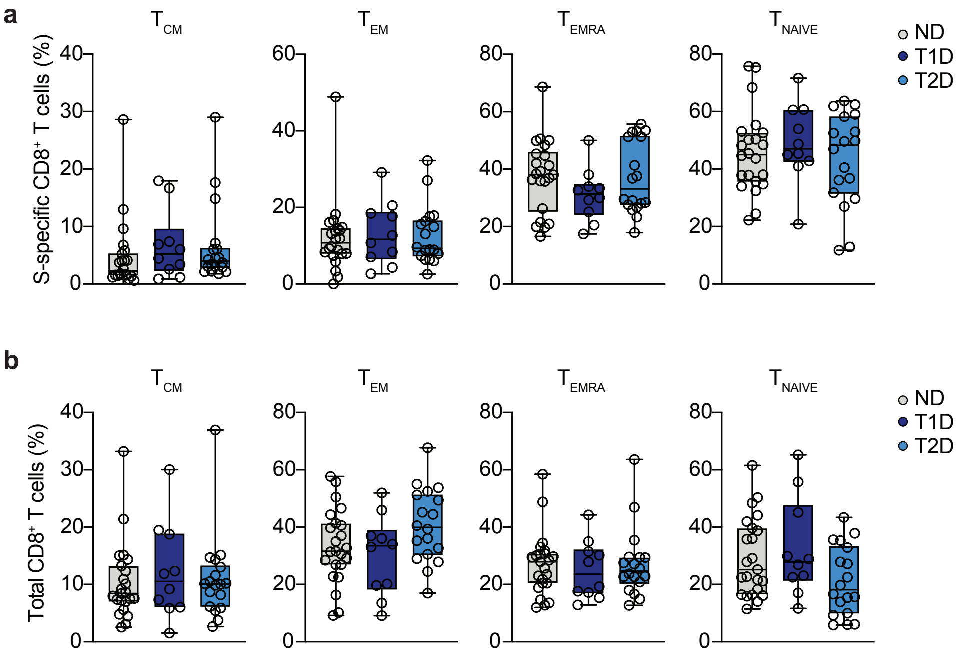


**Supplementary Figure 4. Antigen experience of S-specific (a) and total (b) CD8+ T cells.**

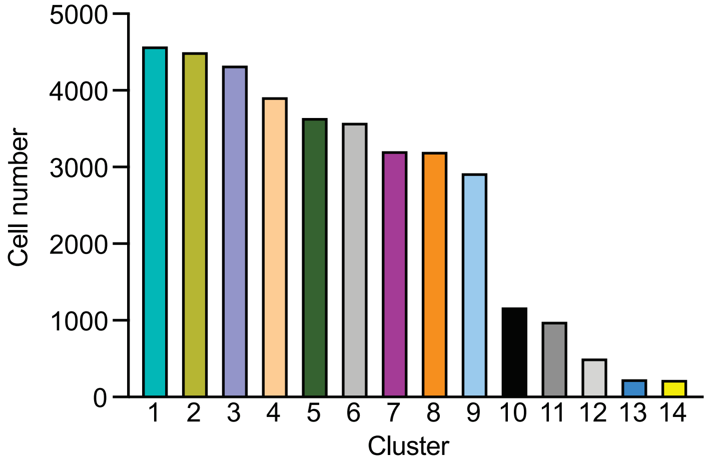


**Supplementary Figure 5. Cell numbers in S-specific CD8⁺ T cell cluster analysis**

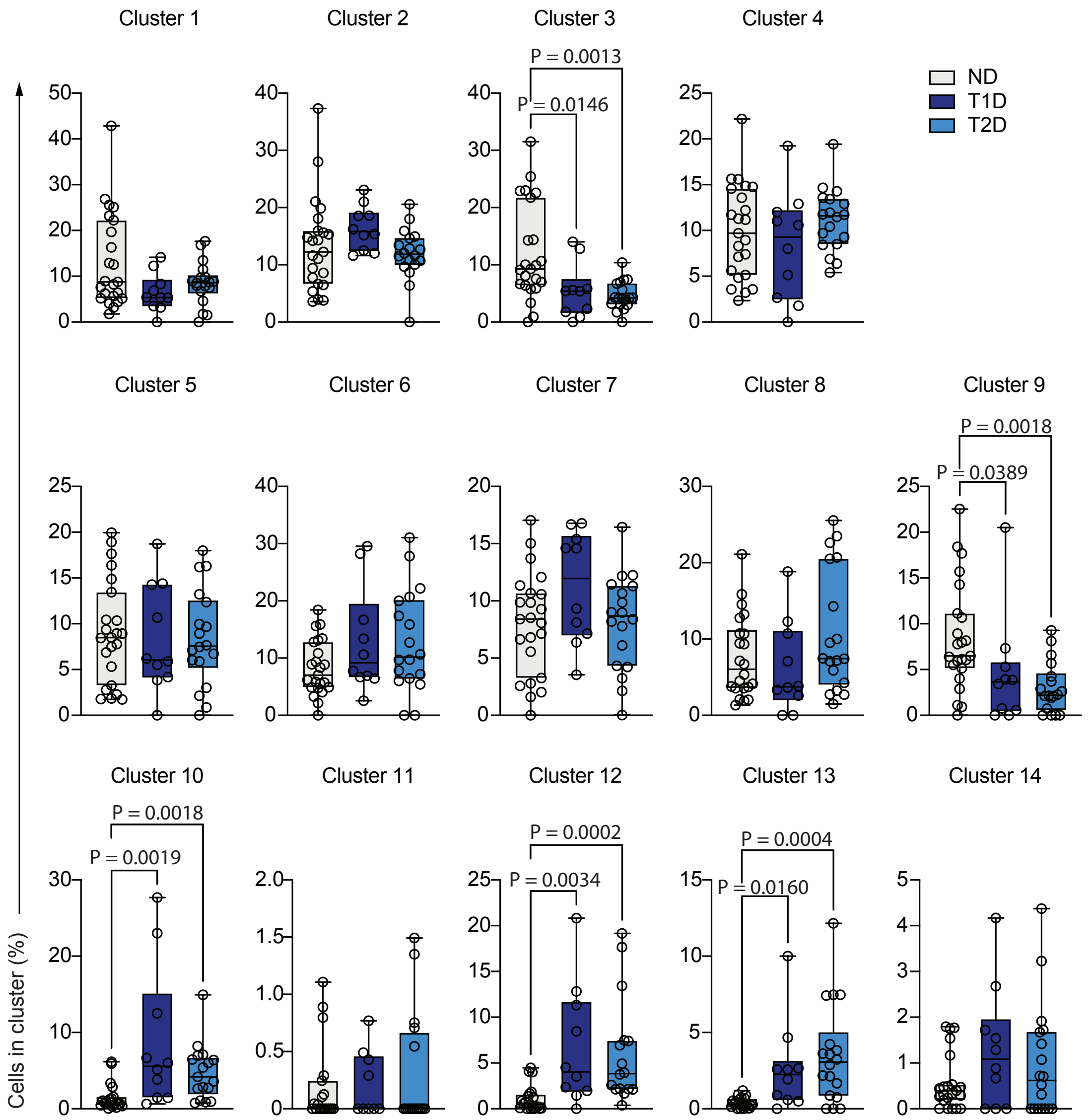


**Supplementary Figure 6. S-specific CD8⁺ T cell cluster distribution.** Percentage of S-specific CD8⁺ T cells in each of the identified clusters. One-way ANOVA with Tukey's correction for multiple comparisons.

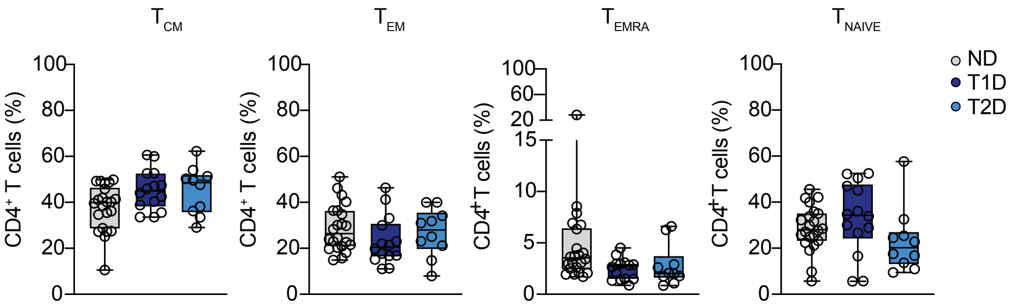


**Supplementary Figure 7. Antigen experience in CD4⁺ T cell recall responses**. Summary of percentage of total CD4⁺ T cells from ND controls, T1D and T2D participants with a naïve, memory, central memory or effector memory cells re-expressing CD45RA phenotype. One-way ANOVA with Tukey's correction for multiple comparisons.

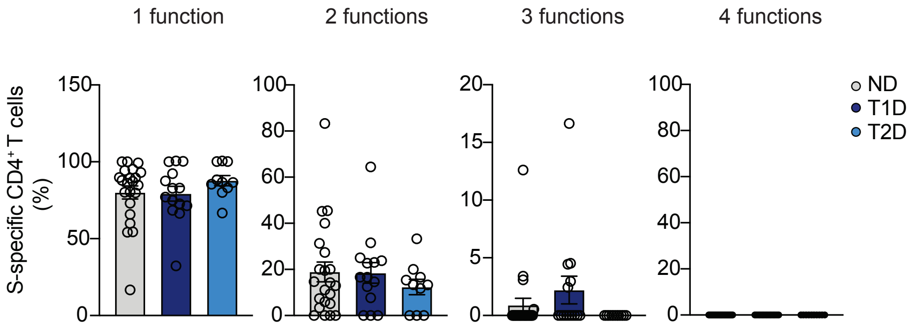


**Supplementary Figure 8. Polyfunctionality of S-specific CD4+ T cells.** Percentage of S-specific CD4$^{+}$ T cells from ND controls, T1D and T2D participants that co-express one, two, three or four cytokines (functions). One-way ANOVA with Tukey's correction for multiple comparisons.

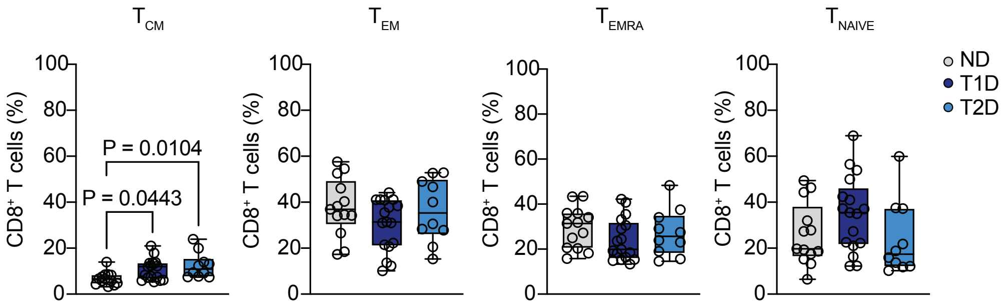


**Supplementary Figure 9. Antigen experience in CD8⁺ T cell recall responses.** Summary of percentage of total CD8⁺ T cell from ND, T1D, and T2D participants with a naïve, memory, central memory or effector memory cells re-expressing CD45RA phenotype. One-way ANOVA with Tukey's correction for multiple comparisons.

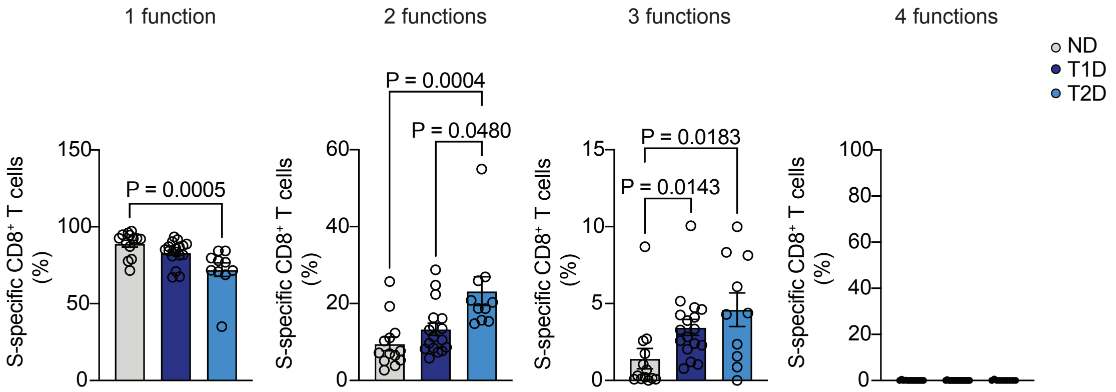


**Supplementary Figure 10. Polyfunctionality of S-specific CD8⁺ T cells.** Percentage of S-specific CD8⁺ T cells from ND controls, T1D and T2D participants that co-express one, two, three or four cytokines (functions). One-way ANOVA with Tukey's correction for multiple comparisons.

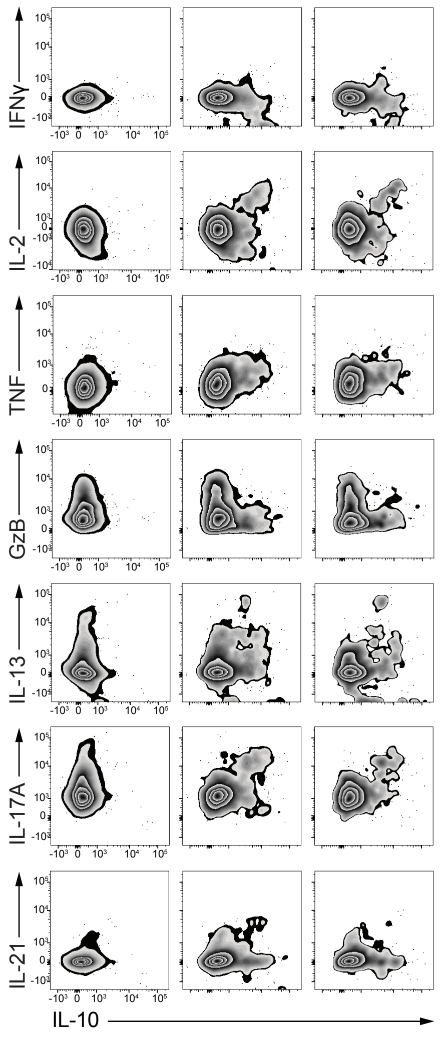


**Supplementary Figure 11. Co-expression of IL-10 by S-specific, cytokine-producing CD8⁺ T cells.** Dot plots show the co-expression of IL-10 and other cytokines in S-specific CD8+ T cells from ND (left), T1D (middle) and T2D (right) individuals.

| | No diabetes | T1D | T2D |
|---|---|---|---|
| Number of participants | 44 | 44 | 46 |
| Median age (min-max) | 52.5 (27-62) | 49 (22-64) | 60 (44-75) |
| Sex | | | |
| Female (%) | 38.6 | 43.2 | 41.3 |
| Male (%) | 61.4 | 56.8 | 58.7 |
| Median BMI (mix-max) | 24 (18-36) | 25 (18-41) | 32 (19-43) |
| Average diabetes duration in years (min-max) | N/A | 22 (0-49) | 14 (1-41) |
| | | | |
| Vaccine type | | | |
| BNT162b2 (Pfizer/BioNTech) | 12 | 39 | 38 |
| mRNA-1273 (Moderna) | 32 | 3 | 5 |
| ChAdOx1-S (Oxford/Astra Zeneca) | 0 | 2 | 3 |

**Supplementary Table 1. Participant characteristics.**

vaccine-specific CD4 cell numbers/participant

| ND | T1D | T2D |
|---|---|---|
| 9 | 47 | 35 |
| 2352 | 13 | 19 |
| 1702 | 50 | 22 |
| 83 | 75 | 125 |
| 312 | 193 | 124 |
| 6 | 185 | 185 |
| 9 | 997 | 1381 |
| 44 | 33 | 32 |
| 129 | 104 | 23 |
| 262 | 57 | 12 |
| 226 | 79 | 28 |
| 122 | 672 | 106 |
| 1071 | 37 | 110 |
| 240 | 373 | 26 |
| 419 | | 62 |
| 595 | | 24 |
| 412 | | 307 |
| 534 | | 68 |
| 151 | | 87 |
| 6 | | 366 |
| 182 | | 46 |
| 134 | | 270 |
| 1595 | | |
| 269 | | |
| 234 | | |
| 506 | | |

vaccine-specific CD8 cell numbers/participant

| ND | T1D | T2D |
|---|---|---|
| 2400 | 345 | 31 |
| 271 | 24 | 115 |
| 4078 | 39 | 17 |
| 2294 | 130 | 134 |
| 3742 | 155 | 280 |
| 108 | 112 | 261 |
| 225 | 407 | 636 |
| 376 | 150 | 283 |
| 975 | 466 | 74 |
| 2706 | 113 | 116 |
| 56 | | 133 |
| 423 | | 239 |
| 446 | | 67 |
| 1866 | | 189 |
| 343 | | 139 |
| 2940 | | 141 |
| 812 | | 183 |
| 56 | | 47 |
| 1592 | | |
| 350 | | |
| 514 | | |
| 3779 | | |
| 1683 | | |

**Supplementary Table 2. S-specific T cell numbers for clustering analysis.**
